# Human forebrain endothelial cell therapy for psychiatric disorders

**DOI:** 10.1038/s41380-020-0839-9

**Published:** 2020-07-13

**Authors:** Debkanya Datta, Sivan Subburaju, Sarah Kaye, Jugajyoti Baruah, Yong Kee Choi, Yeqi Nian, Jahan S. Khalili, Sangmi Chung, Abdallah Elkhal, Anju Vasudevan

**Affiliations:** 1 Angiogenesis and Brain Development Laboratory, Huntington Medical Research Institutes (HMRI), 686 S Fair Oaks Avenue, Pasadena, CA 91105 USA; 2 Department of Psychiatry, Harvard Medical School, Boston, MA 02215 USA; 3 Division of Basic Neuroscience, McLean Hospital, 115 Mill Street, Belmont, MA 02478 USA; 4 Department of Surgery, Harvard Medical School, Boston, MA 02115 USA; 5 Division of Transplantation, Brigham and Women’s Hospital, 221 Longwood Avenue, EBRC 309, Boston, MA 02115 USA; 6 Personal Peptides LLC, Houston, TX 77002 USA; 7 Department of Cell biology and Anatomy, New York Medical College, Valhalla, NY 10595 USA

**Keywords:** Neuroscience, Stem cells, Cell biology, Psychiatric disorders

## Abstract

Abnormalities of or reductions in GABAergic interneurons are implicated in the pathology of severe neuropsychiatric disorders, for which effective treatments are still elusive. Transplantation of human stem cell-derived interneurons is a promising cell-based therapy for treatment of these disorders. In mouse xenograft studies, human stem cell-derived-interneuron precursors could differentiate in vivo, but required a prolonged time of four to seven months to migrate from the graft site and integrate with the host tissue. This poses a serious roadblock for clinical translation of this approach. For transplantation to be effective, grafted neurons should migrate to affected areas at a faster rate. We have previously shown that endothelial cells of the periventricular vascular network are the natural substrates for GABAergic interneurons in the developing mouse forebrain, and provide valuable guidance cues for their long-distance migration. In addition, periventricular endothelial cells house a GABA signaling pathway with direct implications for psychiatric disease origin. In this study we translated this discovery into human, with significant therapeutic implications. We generated human periventricular endothelial cells, using human pluripotent stem cell technology, and extensively characterized its molecular, cellular, and functional properties. Co-culture of human periventricular endothelial cells with human interneurons significantly accelerated interneuron migration in vitro and led to faster migration and wider distribution of grafted interneurons in vivo, compared to neuron-only transplants. Furthermore, the co-transplantation strategy was able to rescue abnormal behavioral symptoms in a pre-clinical model of psychiatric disorder, within 1 month after transplantation. We anticipate this strategy to open new doors and facilitate exciting advances in angiogenesis-mediated treatment of psychiatric disorders.

## Introduction

Abnormal migration, positioning, and reduction in GABAergic interneurons during the critical prenatal developmental period results in dysfunctional cortical neuronal synchrony implicated in brain diseases such as autism, epilepsy, and schizophrenia [1–5], conditions awaiting more effective treatments. Cell transplantation is a powerful tool to introduce new cells with intrinsic plasticity to overcome cellular deficits and initiate repair and regeneration. To be successful, grafted cells should possess the ability to migrate and disperse through affected areas, differentiate into fully mature neurons, functionally integrate, and modulate circuitry activity in the damaged host brain. A better comprehension of the cellular and molecular mechanisms of neuronal development has led to use of their precursors in transplantation [6–8]. While the origin and specification of cortical GABAergic interneurons was well established [9–11], mechanisms that underlined their migration were not fully understood. Our studies served to address this critical gap by showing that embryonic forebrain vascular networks are strategically positioned to provide physical support and critical guidance cues for GABAergic interneuron migration in the developing telencephalon [12–14]. In addition, our work has established novel autonomous links between the periventricular vascular network and the origin of psychiatric disorders, from the earliest developmental time points [13, 15]. Understanding brain development thus begins with an appreciation of all of its cellular components. Deeper insights into the anatomy, origin, molecular regulation, function, and dysfunction of the periventricular vascular network in the last decade [12–15] were crucial for understanding its direct significance for psychiatric disorders.

Due to the restricted availability of human fetal tissue for cell therapy, human pluripotent stem cell (hPSC) technology provides an unprecedented opportunity to study disease mechanisms [16–22]. Multiple groups have successfully derived human interneuron/interneuron progenitors from hPSCs [23–26] and transplantation of interneurons/interneuron progenitors has emerged as a promising treatment option for psychiatric disorders [27–32]. When transplanted in mouse [33] and rat [34] models of epilepsy, hPSC-derived-interneuron precursors, survived well, fired action potentials, formed functional synaptic connections and could reduce abnormal seizure activities. Though showing great promise, one issue that needs improvement is the migration efficiency of transplanted cells. At 2 weeks post transplantation, transplanted interneurons displayed minimal migration, and it was only at 4–7 months post transplantation, that some migration and integration into host brain was observed [23, 25, 33, 34]. Therefore, the beneficial effects of interneuron graft-in-disease models were observed only several months after transplantation. This presents an obstacle for the clinical translation of interneuron-based therapy, especially for very sick or severely affected patients. Another drawback that has been described with GABA producing cell types after transplantation is their transient effects, due to reductions in GABA levels [35, 36]. A decrease in GABA-mediated inhibition is a critical contributing factor for hyperexcitability and seizure initiation and increased secretion of GABA, by grafted cells, is important for increasing the seizure threshold. Thus, at present, while transplantation of GABAergic interneurons represents the most promising cell-based therapeutic alternative for GABA-related diseases, there are difficulties that need to be overcome.

Our key fundamental discovery that pre-formed vascular networks are the natural guides for GABAergic neuronal migration from the earliest developmental time point assumes a new significance here that can serve to improve hPSC-derived GABAergic neuronal migration. The periventricular vascular network not only acts as a physical substrate for neuronal migration in the embryonic forebrain, but also has a very unique gene expression profile, unlike endothelial cells from other brain regions or organs [12–15]. Periventricular endothelial cells (PVECs) show enriched expression of cell surface marker, GABA_A_ receptor β3 subunit (GABRB3) as opposed to pial endothelial cells or control endothelial cells prepared from midbrain and hindbrain [15]. Therefore, GABRB3 serves as a valuable tool to selectively sort human endothelial cells, which are akin to mouse PVECs. In addition, we know that PVECs express and release GABA that promotes rapid and extensive long-distance migration of GABAergic interneurons [15]. Neuronal GABA cannot compensate for the roles of endothelial GABA and interneurons stall in their migration in the absence of endothelial GABA [15]. This close neurovascular interaction during embryonic brain development that is sealed by space and time is the key missing link in interneuron-based therapy. Interestingly, it has been reported that transplanted neuronal precursors align and migrate along the surface of host blood vessels [37, 38] as though in search of a missing or lost counterpart. All of this fundamental knowledge provided us with strong rationale to generate human PVECs from human embryonic stem cells and to tap into the potential of these endothelial cells to improve human GABAergic interneuron migration in vitro and in vivo.

Our results show that human PVECs can faithfully mimic the functional aspects of mouse periventricular angiogenesis and promote long-distance migration of human GABAergic interneurons in culture and after transplantation into multiple mouse brain regions. In addition, this co-transplantation approach markedly improved cell migration and dispersion, stabilized GABA release levels and improved behavioral outcome in a pre-clinical model of psychiatric disorder, within 1 month after transplantation, as opposed to interneuron-only transplantation, which did not show any rescue in the same time frame. Notably, this work shows for the first time how prenatal forebrain angiogenesis, when tapped into correctly, has a remarkable potential for repair and regeneration in the adult brain that can serve to ameliorate abnormal psychiatric behaviors.

## Results

### Efficient generation of human PVECs from human embryonic stem cells

**Fig. 1:**
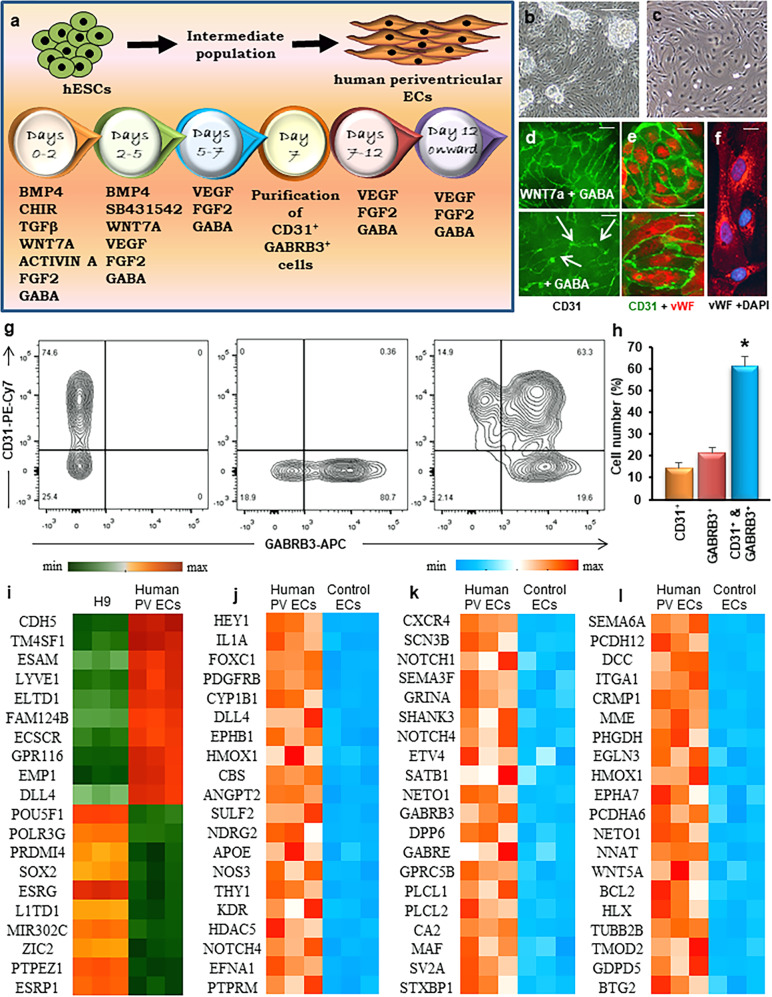
Derivation of human periventricular endothelial cells from human embryonic stem cells. **a** Schematic of the human periventricular endothelial cell differentiation protocol. Phase contrast images of the day 6 cell population (**b**) and pure population of human periventricular endothelial cells at day 10 (**c**). **d** Periventricular endothelial cells showed robust CD31 expression upon addition of WNT7A and GABA from day 0–5 of differentiation, while addition of GABA alone, selectively increased CD31 expression on endothelial cell–cell junctions. **e** Co-labeling with endothelial cell markers, vWF (red) and CD31 (green) of human periventricular endothelial cells. **f** High-magnification image of vWF labeling of periventricular endothelial cells. **g** Flow cytometry analysis showing CD31^+^GABRB3^−^, CD31^−^GABRB3^+^ and double positive CD31^+^GABRB3^+^ populations. **h** Frequencies of human periventricular endothelial cells are CD31^+^/GABRB3^+^. Data represent mean ± SD (*n* = 6, *P < 0.05, Student’s *t* test). **i** Heatmap showing top 20 differentially expressed genes in H9 cells versus H9-derived periventricular endothelial cells (PV ECs), indicating efficient endothelial cell differentiation. Heatmaps showing top 20 upregulated genes in H9-derived periventricular endothelial cells versus control endothelial cells in three different categories: angiogenesis (**j**), GABA pathway (**k**) and neurogenesis (**l**) categories. Scale bars: **b** 100 µm (applies to **c**), **d** 50 µm (applies to **e**, **f**).

Brain endothelial cells have been derived from hPSC previously [39–42], but their analysis, gene expression studies and functions have largely focused on the blood–brain barrier attributes. Generation of embryonic forebrain specific PVECs has not been reported so far. PVECs show enrichment in genes controlling neurogenesis, neuronal migration, chemotaxis, and axon guidance and can be distinguished from other endothelial cells by their specific transcription factor expression, signaling molecules and extracellular receptors [12–15]. Indeed, this specific population of endothelial cells houses a novel GABA signaling pathway that is distinct from the classical neuronal GABA signaling pathway in the embryonic forebrain. Vasculature is autonomous with respect to anatomy, patterning, gene expression, developmental regulation, and function in different organs and/or regions at different time points. Developing strategies to generate embryonic forebrain-like vasculature will augment its region-specific vessel growth and function after transplantation and will be important for clinical applications. Based on our knowledge of gene expression of mouse PVECs of the embryonic forebrain, we first established a differentiation strategy to generate human PVECs from human ES cell lines (Fig. 1a–c; Supplementary Figs. 1–4). The strategy was initially implemented using the human ES cell line H9, and robustness of the protocol was subsequently validated with the human ES cell line H1. From day 0–2 of differentiation, mesodermal cell fate was induced in H9 cells by culturing them in E8 media supplemented with bone morphogenetic protein 4 (BMP4), Activin A, and CHIR 99021 (small molecule activator of WNT pathway). From days 2–5, vascular induction was promoted by inhibiting TGFβ signaling (using the small molecular inhibitor SB431542) and by addition of vascular endothelial growth factor-A (VEGF-A). Since PVECs express Wnt7a and GABA, we added these components to the differentiating medium. A combination of WNT7A protein and low concentration of GABA (5 μM) from days 0 to 5 in the differentiating medium was critical for tight junction formation and production of endogenous GABA in these endothelial cells (Supplementary Figs. 1 and 2). Interestingly, higher levels of GABA (50 or 100 µM) disrupted the localization of tight junction proteins Claudin 5 and ZO-1, alluding to a correlation between GABA level and tight junction formation in PVECs (Supplementary Fig. 1d–i). Addition of WNT7A and GABA also significantly increased expression of transcription factors *Pax6, Dlx1*, *Dlx2*, *Nkx2.1*, tight junction component *Claudin 5*, *Vegf* receptor *Flk1*, cell-cell adhesion molecule *Cd31* and chemokine 12 or *Cxcl12* (also known as *stromal cell-derived factor 1* or *SDF-1*), and SDF-1 receptor *Cxcr4*, (Supplementary Fig. 3). Immunocytochemistry and western blotting data illustrate the importance of WNT7A and GABA co-addition in the differentiating medium for CD31 and VE-Cadherin expression (Fig. 1d, Supplementary Fig. 4) in PVECs. From day 5 of differentiation, endothelial-like cells were observed in the differentiating cell population that expressed markers CD31 and vWF (Fig. 1e, f). Of importance, on day 7, differentiated CD31^+^GABRB3^+^ cells (>60% of total differentiated cells) were isolated by fluorescence-activated cell sorting (FACS) (Fig. 1g, h) and were further maintained in endothelial cell culture medium containing VEGF, FGF2, and GABA.

To test the efficacy and specificity of our differentiation protocol, we performed microarray analyses of H9 ES cells, human PVECs (Fig. 1i–l; Supplementary Fig. 5) and commercially available human-iPSC-derived human aortic endothelial cell (HAEC)-like cells (see “Materials and methods” and Supplementary Fig. 6) as control. We extracted RNA from cells of these three groups and performed microarray hybridization and analysis. A comparison of gene expression between H9 cells and human PVECs showed a distinct upregulation of angiogenesis related genes in human PVECs only, while H9 cells depicted an upregulation of embryonic stem cell-related genes or markers of pluripotency (Fig. 1i). GSEA analysis also revealed an enrichment of angiogenesis gene sets in only PVECs (Supplementary Fig. 5), indicating the effectiveness of the differentiation. Next, we compared the gene expression of human PVECs with control endothelial cells (Fig. 1j–l). This resulted in an upregulation of 1947 genes and a downregulation of 1873 genes in human PVECs versus controls. Specific angiogenesis-related genes were significantly upregulated in PVECs versus control endothelial cells (Fig. 1j), further indicating the distinct nature of the two endothelial cell types. In addition, gene expression that is specific to the central theme of forebrain development like GABA neuron development (Fig. 1k) and neurogenesis (Fig. 1l) was enriched in PVECs versus control endothelial cells, reflective of PVECs’ unique molecular signature [12–15]. A clear separation between PVECs and control endothelial cells was observed along the PCA axis (Supplementary Fig. 6a) with respect to gene expression (Supplementary Fig. 6b, c) and gene ontology categories depicting biological processes (Supplementary Fig. 6d). Fate mapping further confirmed the cardiac fate of control endothelial cells (Supplementary Fig. 6e). A violin plot portrays differential gene expression of top genes in several categories (angiogenesis, GABA receptor, transcription factor, tight junction and Wnt signaling) in PVECs versus control endothelial cells (Supplementary Fig. 7). We also compared the gene expression profile of human PVECs with mouse PVECs and found a significant overlap in common blood vessel development and GABA pathway-related genes (Supplementary Fig. 8).

### Cellular and functional characterization of human PVECs

**Fig. 2:**
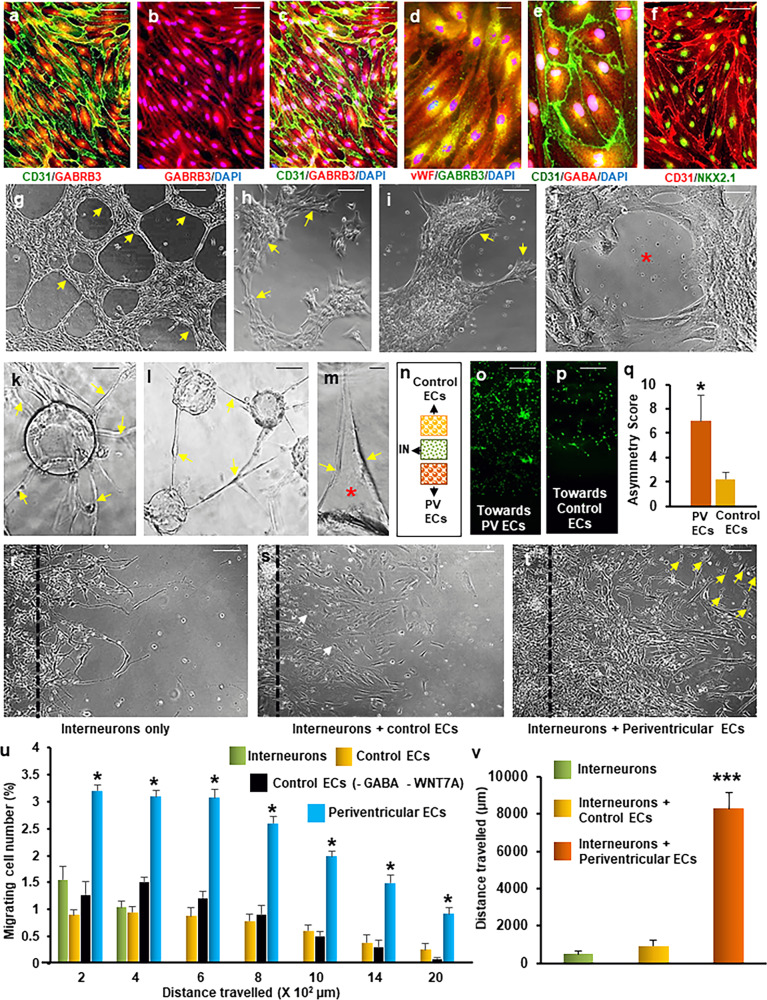
Molecular and functional characterization of H9-derived human periventricular endothelial cells. **a–c** Images of GABRB3 (red) and CD31 (green) expression in all human periventricular endothelial cells and merged images with DAPI. Co-labeled images of vWF (red) and GABRB3 (green) (**d**), CD31 (green) and GABA (red) (**e**), and CD31 (green) and NKX2.1 (red) (**f**) in periventricular endothelial cells. **g** Bright-field image showing the tube-formation (yellow arrows) ability of human periventricular endothelial cells. **h–j** Tube-formation assay in 3D-fibrin gels. When cultured in fibrin gel, human periventricular endothelial cells aligned (yellow arrows) and formed tubular structures with a lumen (red asterisk) within 48 h. Sprouting and tube formation in fibrin gels. Nascent sprouts (yellow arrows) are observed on day 2 (**k**) that continue to proliferate, migrate, and form intercellular tubes (yellow arrows in **l**) with a clear lumen (red asterisk, **m**). **n** Schematic of the chemoattraction assay. Using a three-well culture insert, interneurons (IN) were seeded in the middle (green dotted rectangle), while periventricular endothelial cells (PV ECs; orange dotted rectangle) and control endothelial cells (ECs; yellow dotted rectangle) were seeded on either side. β-tubulin labeled images of interneurons showing robust migration toward periventricular endothelial cells (**o**) but not toward control endothelial cells (**p**). **q** Quantification of chemoattraction of interneurons toward periventricular endothelial cells versus control endothelial cells. Data represent mean ± SD, *(n* = 10, *P < 0.05, Student’s *t* test). Scoring scheme modified from Won et al. [13] Migration of interneurons in interneuron-only culture (**r**), when co-seeded with control endothelial cells (**s**), and co-seeded with periventricular endothelial cells (**t**) 48 h after seeding. The black dotted line represents the day 0 mark. Interneurons when co-seeded with periventricular endothelial cells migrated over a farther distance (shown in yellow arrows in **t**) than compared to when seeded alone (**r**) or seeded with control endothelial cells (shown in white arrows in **s**). **u** Quantitation of long-distance migration ability of human periventricular endothelial cells, in comparison to interneurons, control endothelial cells (HAEC-like) and endothelial cells derived without GABA and WNT7A at 48 h post seeding. Robust migration of periventricular endothelial cells over long distance was observed. Data represent mean ± SD, (*n* = 10, ***P < 0.001, *P < 0.05, Student’s *t* test). **v** Quantification of distance migrated by interneurons when seeded alone, co-seeded with control endothelial cells, and co-seeded with periventricular endothelial cells. Graph shows the distance traveled by the 50 farthest interneurons in each condition on day 5. Migration of interneurons was significantly accelerated when co-seeded with periventricular endothelial cells. Data represent mean ± SD, (*n* = 10, ***P < 0.001, Student’s *t* test). Scale bars: **a** 100 µm (applies to **b**, **c**, **f**, **g**-**l**, **o**, **p**, **r**–**t**), **d** 50 µm (applies to **e**, **m**).

We further assessed the expression of PVEC markers in purified human PVEC cultures by immunocytochemistry. All cells in culture (100%) expressed endothelial cell markers CD31, (Fig. 2a, c, e, f; Supplementary Figs. 9 and 10), von Willebrand Factor (vWF) (Fig. 2d), and co-labeled with PVEC markers, GABRB3 (Fig. 2a–c; Supplementary Fig. 9), GABA (Fig. 2e; Supplementary Fig. 9), NKX2.1 (Fig. 2f; Supplementary Fig. 9), PAX6, and ISL1 (Supplementary Fig. 10) similar to mouse PVECs. Pluripotent markers OCT4 and TRA1-60 were not present in any cell, confirming the absence of undifferentiated cells in the PVEC population (Supplementary Fig. 11). Effective proliferation, migration, sprouting, alignment, branching, lumen formation, and anastomosis are key elements of angiogenesis. Therefore, we performed in vitro assays to demonstrate the angiogenic properties of human PVECs. These endothelial cells formed tubular networks within 24 h of seeding on Matrigel (Fig. 2g), demonstrating high angiogenic capacity. In addition, human PVECs formed tubular networks in a three-dimensional milieu when cultured within fibrin gel matrix demonstrating budding, branching, and lumen formation (Fig. 2h–j). Later stages of endothelial cell branching and fusion of vessels (anastomosis) were also observed (Fig. 2h–j). Sprouting of new capillaries from existing blood vessels is another hallmark of angiogenesis. We performed a fibrin gel bead assay for sprouting, in which PVECs were coated on cytodex beads and embedded in three-dimensional fibrin gels. PVECs started budding and sprouting within 2 days (Fig. 2k). Within the next days, long tubular vessels with clear intercellular lumens were formed (Fig. 2l, m). In addition to general angiogenic properties, PVECs possess some unique properties. First, PVECs themselves have the ability to migrate long distance. Second, PVECs induce long-distance migration of GABAergic interneurons. We tested these properties in human PVECs using in vitro chemoattractivity and migration assays. We used human GABAergic interneurons (Cellular Dynamics; Supplementary Fig. 12) that have been extensively characterized with respect to their morphological, electrophysiological and molecular characteristics in several neuroscience research models, as a source of neurons for these assays. Using three-well culture inserts (ibidi GmbH), we seeded human interneurons in a small rectangular patch on a 35 mm poly-ornithine/laminin coated culture dish. Equal number of PVECs and control HAEC-like endothelial cells (that do not have periventricular-specific gene expression; Fig. 1j–l; Supplementary Figs. 6–8) were seeded as patches on either side of the neuronal patch, with the gap between each patch being 500 µm (Fig. 2n). The number of interneurons that migrated toward PVECs versus control endothelial cells was quantitated after 36 h. Interneurons showed significantly higher chemo-attractive response toward PVECs compared to control endothelial cells (Fig. 2o–q), confirming that human interneurons respond selectively to chemo-attractive cues secreted by human PVECs. Next, we performed cell migration assays to test the long-distance migratory potential of human PVECs and its role in guiding human interneuron migration (Fig. 2r–v). We compared the migration of PVECs with human interneurons, and with two types of control endothelial cells: (a) HAEC-like human endothelial cells and (b) endothelial cells derived from H9 cells in the absence of GABA and WNT7A (Supplementary Figs. 2–4). Human PVECs seeded alone, traveled further distance compared to human interneurons or either set of control endothelial cells (Fig. 2u), confirming their cell-intrinsic capacity for long-distance migration. Also, for the same distance range, a higher percentage of PVECs migrated out than interneurons or control endothelial cells (Fig. 2u). To test the ability of human PVECs in facilitating human interneuron migration, we performed a co-culture migration assay where interneurons were co-seeded with human PVECs. Migration of co-seeded interneurons were compared to interneurons, which were seeded alone, or seeded along with control human endothelial cells. When seeded along with PVECs, interneurons migrated significantly, both in terms of cell number and distance when compared to interneurons cultured along with control endothelial cells or interneurons alone (Fig. 2r–t, v). Collectively, these data confirm the high angiogenic potential of human PVECs and its specificity with regard to instructing and promoting migration of human GABAergic interneurons.

### Co-transplantation of human PVECs with GABAergic interneurons successfully enhanced neuronal migration in vivo

**Fig. 3:**
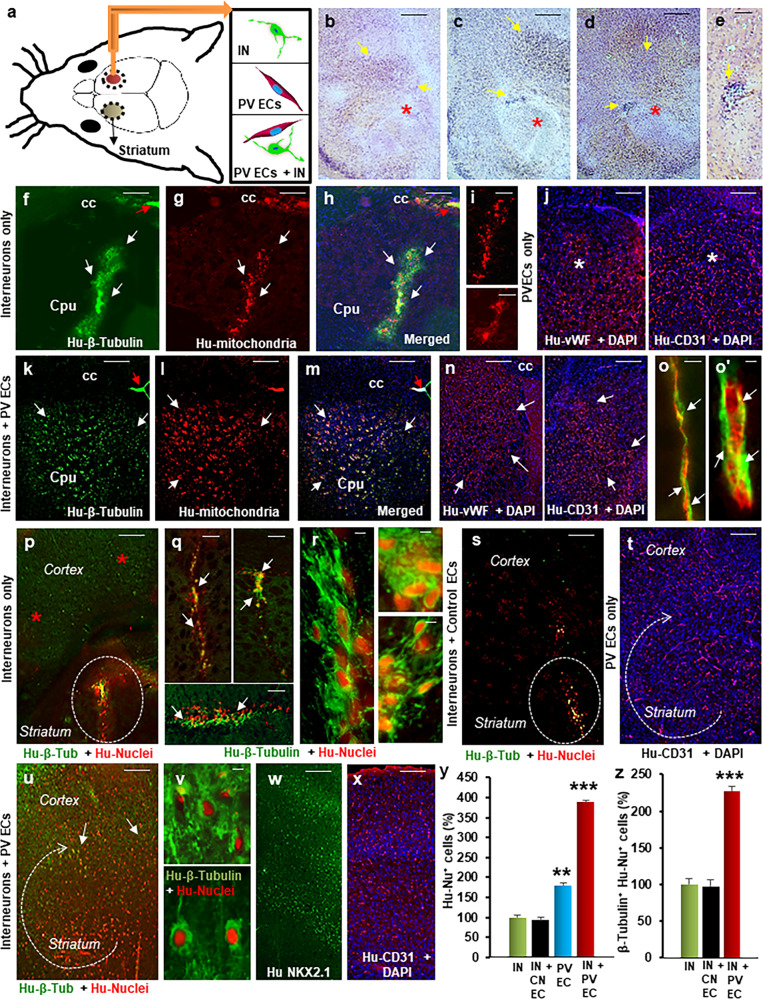
Human periventricular endothelial cells significantly promoted interneuron migration in vivo. **a** Schema of transplantation strategy in adult NOD-SCID mouse. Human interneurons plus periventricular endothelial cells were co-transplanted in the striatum of 8-week-old adult NOD-SCID mice. Interneurons only, periventricular endothelial cells-only, or interneurons plus control endothelial cells were also transplanted in separate experiments. One month after transplantation, brains were collected and processed for histology or IHC. **b–e** H&E staining of co-transplanted brain showing the graft core (red asterisk) and the cell distribution close to graft (yellow arrows). Individual markers human β-tubulin (**f**), human mitochondria (**g**) and a co-labeled image of both with DAPI (**h**) illustrate grafted neurons in interneuron-only transplanted brain that remained mostly within the graft area (white arrows). Red arrow marks lateral ventricle boundary. **i** Magnified images of stalled neurons that are human mitochondria^+^ at graft sites. **j** In periventricular endothelial cells-only transplanted brains, grafted endothelial cells labeled with human vWF and CD31 markers showed robust migration within the striatum. Nuclei are stained with DAPI. **k**–**m** In interneuron + periventricular endothelial cell co-transplanted brains, interneurons showed significantly higher migration ability, and distributed widely in the striatum (white arrows). A co-labeled image of human β-tubulin, human mitochondria and DAPI is shown in m. Red arrows mark the lateral ventricle boundary. **n** Robust migration (white arrows) of periventricular endothelial cells in co-transplanted striatum. (**o**, **o**′) Co-labeling with isolectin B4 and anti-human CD31 shows how newly formed CD31^+^ vessels anastomose with host vessels. **p** In interneuron-only transplanted brains, grafted neurons showed very little migration into the cortex (red asterisks). The white dotted area marks the grafted site in the striatum where most of the neurons remained restricted. Additional stalled neurons (white arrows) from different brains are shown at ×20 (**q**) and ×40 (**r**) magnifications. **s** In neurons + control endothelial cells (without GABA and WNT7A) transplanted brain, grafted neurons remain stalled at the graft site (marked by white dotted circle). **t** In periventricular endothelial cells-only transplanted brain, endothelial cells migrated efficiently into the cortex. **u** In co-transplanted brains, grafted interneurons showed robust migration into cortical region (white arrows). **v** Magnified images (×40) of grafted human interneurons in the cortex. **w** Migrated cells in the cortex of co-transplanted brains show human-NKX2.1 labeling. **x** Robust distribution of human-CD31^+^ endothelial cells in the cortex of co-transplanted brains. **y** Cell count analyses of human-nuclei^+^ cells that have migrated into the cortex after 30 days. Data represent mean ± SD, (*n* = 20, ***P < 0.001, **P < 0.01, Student’s *t* test). **z** Quantification of β-tubulin^+^/human nuclei^+^ neurons in the cortex of transplanted brains 30 days after grafting. Data represent mean ± SD, (*n* = 20, ***P < 0.001, Student’s *t* test). Significantly higher percentage of interneurons in neuron + periventricular endothelial cell co-transplanted brains showed long-distance migration compared to those in interneuron-only condition or interneuron + control endothelial cells derived without GABA and WNT7A condition. For (**y**) and (**z**), cell numbers in cingulate, motor, somatosensory and piriform cortex at all bregma levels were analyzed. Scale bars: **b** 100 µm (applies to **b**–**d**, **f**–**h**, **j**, **k**–**n**, **p**, **s**–**u**, **w**, **x**), **e** 50 µm (applies to **i**, **o**, **q**), **r** 25 µm (applies to **o**′, **v**). Cpu striatum, cc corpus callosum, IN interneurons-only, CN EC: control endothelial cells derived without GABA and WNT7A, PVECs periventricular endothelial cells.

Next, we investigated whether human PVECs could facilitate human interneuron migration in vivo. To this end, we transplanted human PVECs along with human interneurons in a ratio of 1:1 into the striatum of adult NOD-SCID mice, on each side of the brain (Fig. 3a). The striatum provides a potentially powerful experimental system for studying cell migration and dispersion, with optimal cell survival after transplantation. One month after transplantation, histological staining was used to identify the graft (Fig. 3b–e). Many cells were observed migrating out of the graft core into the host brain. To further investigate the migration pattern, we performed immunohistochemistry (IHC) and analysis in 1-month-old PVEC-interneuron co-transplanted brains. The staining patterns were compared to control NOD-SCID brains that had received (1) interneuron-only transplant, (2) PVECs-only transplant, and (3) interneuron + control endothelial cells that were derived without GABA and WNT7A. Grafted interneurons, double labeled with anti-human β-tubulin, anti-human mitochondria and anti-human nuclei antibodies, revealed significant difference in migration pattern between transplanted brains. In interneuron-only and interneuron + control endothelial cell brains, most of the interneurons stayed within the graft site (Fig. 3f–i, s). In contrast, in PVECs-only brain (Fig. 3j) and interneurons along with PVECs co-transplanted brain (Fig. 3k–m), cells migrated out from the graft site and spread widely in the striatum. Human PVECs were identified by anti-human vWF and anti-human CD31 labeling (Fig. 3j, n, t, x). Labeling with anti-human CD31 and isolectin B4 revealed that new vessels formed by human PVEC transplantation were able to merge with host vessels (Fig. 3o, o′). In the interneuron-only group and interneuron + control endothelial cell-transplanted group, the majority of neurons remained stalled near the striatal graft site and only a limited number of grafted neurons were observed in the cerebral cortex (Fig. 3p–s), indicative of failure of long-distance migration. Strikingly, in both the human PVEC group (Fig. 3t) and in the interneuron + PVEC co-transplanted group (Fig. 3u–x), cells from the striatum were able to migrate long distance into several cortical areas, highlighting the enhanced migratory ability of human PVECs alone and along with interneurons. Immunostaining with anti-human-NKX2.1 antibody indicated that grafted interneurons and PVECs continue to express NKX2.1 in the host brain (Fig. 3w). We quantified the percentage of human nuclei^+^ cells, among total grafted cells, that had migrated into the cortex in all four transplanted groups. The cortex of interneuron plus PVECs transplanted brains had a significantly higher number of human nuclei^+^ cells than either of the other groups (Fig. 3y). Interestingly, PVECs-only cortex had almost double the number of grafted cells than interneuron-only cortex, showing that PVECs have a better migratory ability than interneurons or control endothelial cells in vivo (Fig. 3y). Quantification of the neuronal number showed a significant increase in the number of interneurons in the cortex of interneuron + PVEC co-transplanted brains compared to interneuron-only brains and interneuron + control endothelial cell-transplanted brains (Fig. 3z). Cell death was minimal, as confirmed by double staining with anti-caspase and anti-human nuclei antibodies in all of our striatal transplantations (Supplementary Fig. 13). Taken together, these results show that human interneurons require human PVECs for long-distance migration in vivo.

### Human PVEC-derived GABA regulates cell migration

**Fig. 4:**
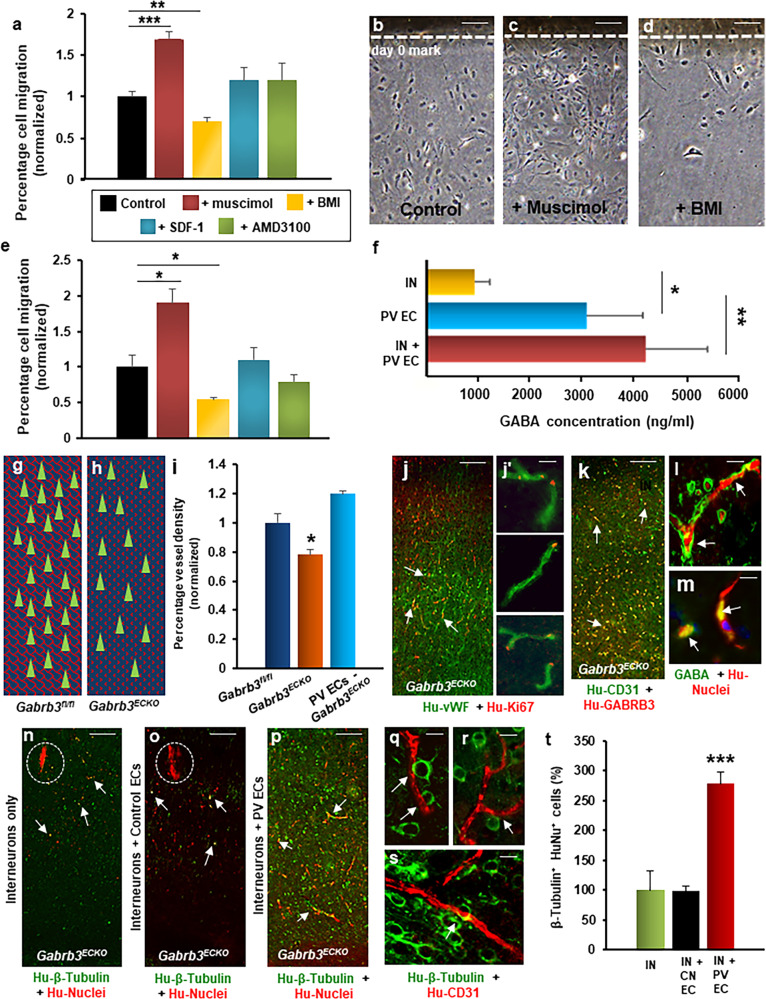
Human periventricular endothelial cell-derived GABA regulates cell migration. **a–d** Migration of periventricular endothelial cells in response to addition of GABA_A_ receptor agonist muscimol (100 µM), GABA_A_ receptor antagonist BMI (100 µM), chemokine SDF-1α (40 nM) and CXCR4 inhibitor AMD3100 (50 µM). **a** Quantitation of migration assay. Addition of muscimol resulted in higher percentage of cells migrating out, while addition of BMI resulted in less cell migration, compared to control (no chemicals added). The presence of SDF-1 or AMD3100 showed no significant effect on migration of periventricular endothelial cells (*n* = 5, mean ± SD, **P < 0.01, ***P < 0.001, Student’s *t* test). **b–d** Representative phase contrast images showing periventricular endothelial cell migration in control, +muscimol, and +BMI conditions. **e** Quantitation of interneuron migration when co-cultured with periventricular endothelial cells pre-treated with chemicals for 48 h. Interneurons co-cultured on muscimol-treated periventricular endothelial cells showed increased migration than those co-cultured on untreated periventricular endothelial cells (control), while interneurons co-cultured with BMI-treated periventricular endothelial cells showed decreased migration compared to control (*n* = 5, mean ± SD, *P < 0.05, Student’s *t* test). Treatment of periventricular endothelial cells with SDF or AMD3100 had no effect on interneuron migration. **f** Quantitation of GABA secretion measured by ELISA. GABA level was significantly higher in interneuron + periventricular endothelial cell co-culture and in periventricular endothelial cell-only population, compared to interneuron-only population. (*n* = 6, mean ± SD, *P < 0.05, **P < 0.01, Student’s *t* test). Schema depicting normal vasculature (red lattice pattern) and GABAergic interneurons (green triangles) in *Gabrb3*^*fl/fl*^ cerebral cortex (**g**), while in *Gabrb3*^*ECKO*^ cerebral cortex (**h**) there is both a vascular deficit (dotted red pattern) and a deficit in GABAergic interneurons (green triangles). **i** Quantification of vessel densities. Periventricular endothelial cell-transplanted *Gabrb3*^*ECKO*^ mice showed significant rescue in vessel densities in the cerebral cortex when compared to *Gabrb3*^*ECKO*^ mice (*n* = 6, mean ± SD, *P < 0.05, Student’s *t* test). Transplanted human periventricular endothelial cells undergo proliferation as shown by human vWF/Ki67 co-labeling (**j**; high magnification images shown in **j**′), and continue to express GABRB3 (**k**) and GABA (**l**, **m**) in vivo (white arrows). (**n–o**) Migration pattern of transplanted interneurons in somatosensory cortical region of transplanted *Gabrb3*^*ECKO*^ brain. Grafted interneurons showed poor migratory ability (white arrows) and remained mostly near the grafted site (dotted white area) in interneuron-only transplanted brain (**n**) and interneuron + control endothelial cell co-transplanted brain (**o**). **p** In interneuron + periventricular endothelial cell co-transplanted brains, interneurons showed extensive migration and widespread distribution (white arrows). **q–s** Magnified images showing close interactions between transplanted interneurons and new vessels formed by human periventricular endothelial cells. **t** Quantitation of β-tubulin+/human nuclei+ neurons in cortex (including cingulate, motor, somatosensory and piriform cortical regions) of *Gabrb3*^*ECKO*^ transplanted brain, 30 days after grafting. Significantly higher number of grafted neurons in interneuron + periventricular endothelial cell co-transplanted *Gabrb3*^*ECKO*^ brains showed migration all over cortex, compared to those in interneuron-only or interneuron + control endothelial cell-transplanted brains. Data represent mean ± SD, (*n* = 20, ***P < 0.001, Student’s *t* test). Scale bar: **b** 100 μm (applies to **c**, **d**, **j**, **k**, **n**–**p**), **l** 50 µm (applies to **j**′ **m**, **q**, **r**, **s**). IN interneurons, CN EC control endothelial cells derived without GABA and WNT7A, PV ECs periventricular endothelial cells.

GABA secreted from mouse PVECs plays both autocrine and paracrine roles. It enhances the migration of PVECs, triggers angiogenesis, and promotes robust migration of interneurons [13, 15]. We next studied whether GABA signaling from human PVECs also performed these functions (Fig. 4a–f). GABA expression in human PVECs was qualitatively confirmed by immunostaining (Fig. 2e; Supplementary Fig 2b and 9h). In addition to expressing GABA, microarray analysis showed high-level expression of chemokine receptor CXCR4 in human PVECs. CXCR4 and its ligand Stromal Derived Factor-1 (SDF-1, also known as CXCL12) regulate cell migration in many developmental events. Therefore, we focused on both GABA and SDF-1/CXCR4 signaling pathways to delineate their role in the migratory events. To study their function in human PVEC migration, we performed a long-distance migration assay with endothelial cells in the presence of GABA_A_ receptor agonist muscimol (100 µM), GABA_A_ receptor antagonist bicuculline methiodide (BMI, 100 µM), human SDF-1 (40 nM), and CXCR4 receptor antagonist AMD3100 (50 µM). There was a significant increase in the number of migrating cells in the muscimol-treated condition compared to control (no chemicals added) (Fig. 4a–c). Correspondingly, there was a significant decrease in cell migration in BMI-treated cells compared to control (Fig. 4a, d). Addition of SDF-1 or AMD3100 produced no significant effect on endothelial cell migration (Fig. 4a). Next, we investigated the effect of these molecules on PVEC-mediated interneuron migration. PVECs in culture were treated with the same agonists and antagonists for 48 h. Interneurons were then seeded over these pre-treated endothelial cells and allowed to migrate for 48 h. Interneurons co-cultured on muscimol-treated endothelial cells showed significantly higher migratory ability compared to control, while interneurons co-cultured with BMI-treated endothelial cells showed decreased migration (Fig. 4e). No discernible effect on interneuron migration was observed in SDF-1 or AMD3100-treated conditions when compared to control (Fig. 4e). These results indicate that the SDF-1/CXCR4 signaling pathway may play other roles in periventricular angiogenesis, independent of cell migration. Using ELISA, we quantitatively analyzed GABA levels secreted from human PVECs and compared it with levels secreted from cultures of human interneurons and co-culture of both these cell types. Human periventricular cells secreted higher levels of GABA than human interneurons, while the co-culture of human PVECs and interneurons secreted the highest levels of GABA (Fig. 4f). Taken together these results show that, activation of the GABA_A_ receptor pathway in human PVECs triggers GABA release that enhances endothelial cell migration, and mediates endothelial cell-guided chemoattractivity and migration of human interneurons.

### Rescue of behavioral symptoms in *Gabrb3*^*ECKO*^ mice in 4 weeks

In order to test the functional significance of co-transplantation of human PVECs with interneurons, when compared to interneuron-only transplants, we used a well-established pre-clinical model of psychiatric disorder—the *Gabrb3* endothelial cell conditional knockout model (*Gabrb3*^*ECKO*^ mice), in which there are both vascular and GABAergic interneuron deficits in the cingulate, motor, and somatosensory cortex [15]. The developmental dysfunction of endothelial GABA_A_ receptors and reduction in endothelial GABA levels significantly impaired angiogenesis and GABAergic interneuron migration in the embryonic brain that persisted in the adult brain (Fig. 4g, h) with lasting consequences for behavioral outcome [15]. These mice show behavioral dysfunction similar to psychiatric disease that is characterized by these core symptoms—depression, increased anxiety, communication deficits, impaired social recognition, and reduced social interactions. Therefore, we transplanted interneurons only or co-transplanted human interneurons and PVECs into the somatosensory cortex of *Gabrb3*^*ECKO*^ mice. As additional control groups, we transplanted human PVECs only and interneurons + control endothelial cells (derived without GABA and WNT7A) into the *Gabrb3*^*ECKO*^ somatosensory cortex. Grafted PVECs showed widespread distribution in the *Gabrb3*^*ECKO*^ cortex, rescued vascular densities (Fig. 4i), underwent cell proliferation (Fig. 4j), and continued to express GABRB3 and GABA in vivo (Fig. 4k–m). Immunostaining of grafted interneurons in interneuron-only and interneuron + control endothelial cell co-transplanted brains showed that neurons exhibited limited migration and remained close to the transplantation site with poor migration and distribution (Fig. 4n, o, t; Supplementary Fig. 14), similar to our observations in the interneuron-only and interneuron + control endothelial cell transplantations in the striatum (Fig. 3f–i, p–s). In sharp contrast, interneuron + PVECs co-transplanted brains showed widespread migration of grafted interneurons in the entire cortical area at 1 month post transplantation (Fig. 4p, q–t; Supplementary Fig. 14).

**Fig. 5:**
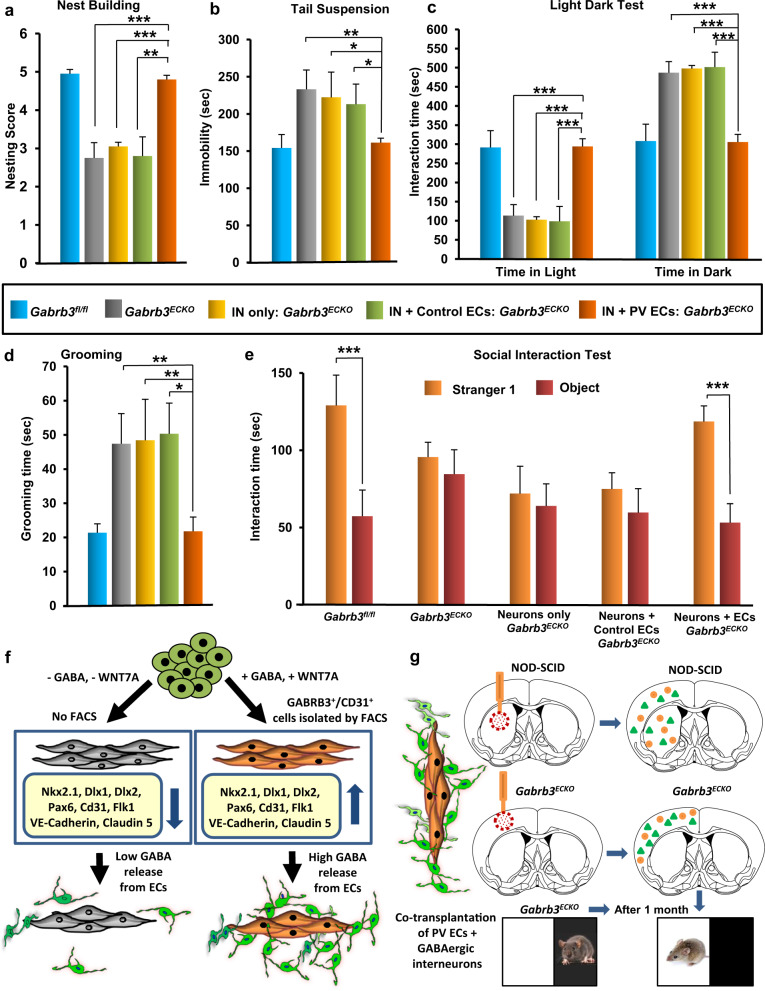
Co-transplantation of human periventricular endothelial cells with human interneurons in cortex ameliorates behavioral abnormalities in *Gabrb3*^*ECKO*^ mice. **a**–**e** Quantification of behavioral tests performed in five different groups of mice: 1) sham *Gabrb3*^*fl/fl*^*, 2) sham  Gabrb3*^*ECKO*^, 3) interneurons only transplanted into the cortex of *Gabrb3*^*ECKO*^ mice, 4) interneurons + control endothelial cells derived without GABA and WNT7A co-transplanted (1:1 ratio) into the cortex of *Gabrb3*^*ECKO*^ mice and 5) interneuron + periventricular endothelial cells co-transplanted (1:1 ratio) into the cortex of *Gabrb3*^*ECKO*^ mice. In all transplanted and sham surgery mice, tests were done 30 days post transplantation. In each case, data represent mean ± SD, (*n* = 6, *P < 0.05, Student’s *t* test). **a** Nesting behavior was assayed by a five-point nesting score. Interneuron + periventricular endothelial cell co-transplanted mice showed normal nesting behavior, similar to sham control mice. Interneuron-only transplanted mice and interneuron + control endothelial cell co-transplanted mice showed poor nest building abilities, similar to *Gabrb3*^*ECKO*^ mice. **b** Tail suspension test. Interneuron + periventricular endothelial cell co-transplanted *Gabrb3*^*ECKO*^ mice showed an immobility time comparable to sham control mice, while interneuron-only transplanted mice and interneurons + control endothelial transplanted mice showed longer immobility time, similar to *Gabrb3*^*ECKO*^ mice. **c** Quantification of exploration time in light–dark box test. Interneuron + periventricular endothelial cell co-transplanted *Gabrb3*^*ECKO*^ mice spent similar time exploring light and dark sides of the box, as observed in sham control mice. Interneuron-only transplanted *Gabrb3*^*ECKO*^ mice and interneuron + control endothelial cell co-transplanted *Gabrb3*^*ECKO*^ mice behaved like Gabrb3^*ECKO*^ mice and continued to spend more time in the dark side of the box. **d** Quantification of self-grooming time. Interneuron + periventricular co-transplanted *Gabrb3*^*ECKO*^ mice showed shorter grooming times, comparable to sham control mice. On the other hand, interneuron-only transplanted *Gabrb3*^*ECKO*^ mice and interneuron + control endothelial cell co-transplanted *Gabrb3*^*ECKO*^ mice continued to show long grooming times, similar to that observed for *Gabrb3*^*ECKO*^ mice. **e** In a three-chamber social interaction test, interneuron + periventricular endothelial cell co-transplanted *Gabrb3*^*ECKO*^ mice spent a significantly longer amount of time interacting with a stranger mouse than with an inanimate object, similar to that observed in sham control mice. In contrast, behavior of interneuron only-transplanted *Gabrb3*^*ECKO*^ mice and interneuron + control endothelial cell co-transplanted mice were reminiscent of *Gabrb3*^*ECKO*^ mice. They did not show any specific preference for the stranger mouse and spent similar time exploring both the stranger mouse and an inanimate object. These behavioral tests show that co-transplantation of interneurons with human periventricular endothelial cells led to an effective rescue of behavioral deficits in *Gabrb3*^*ECKO*^ mice after 1 month of transplantation. **f** Summary schema illustrating the importance of WNT7A/GABA addition to the differentiation medium and isolation of GABRB3^+^/CD31^+^ endothelial cells by FACS for the efficient generation of human periventricular endothelial cells, that release high levels of GABA and promote robust neuronal chemoattractivity and migration. **g** Summary schema depicting a novel co-transplantation strategy of human periventricular endothelial cells with human GABAergic interneurons into multiple brain regions in two different mouse models (NOD-SCID and *Gabrb3*^*ECKO*^) that independently confirm robust cell migration and distribution. The benefit of this co-transplantation manifested as an improvement in behavioral function (light–dark box illustrated in schema) in *Gabrb3*^*ECKO*^ mice, signifying the importance of novel angiogenesis-based treatment strategy for psychiatric disorders.

Subsequently, we evaluated the impact of the cell transplantation and migration on behavioral function in adult mice of the interneuron-only transplanted group, interneuron + control endothelial cell-transplanted group and interneuron + PVEC co-transplanted group, 1 month after transplantation. We compared the results with two groups, *Gabrb3*^*ECKO*^ mice and *Gabrb3*^*fl/fl*^ mice with sham surgery (Fig. 5a–e). *Gabrb3*^*ECKO*^ mice show poor nest building abilities. Interneuron + PVEC co-transplanted *Gabrb3*^*ECKO*^ mice significantly improved their nest building ability and were comparable to sham control mice (Fig. 5a, Supplementary Fig. 15), while interneuron-only *Gabrb3*^*ECKO*^ mice and interneuron + control endothelial cell co-transplanted *Gabrb3*^*ECKO*^ mice failed to build proper nests (Fig. 5a). Next, we used the tail suspension test to evaluate transplanted and control mice for depressive behavior. When suspended by their tails, normal mice show escape-oriented movements and struggle to face up. Immobility of the mouse is defined as a depressive state when the mouse has given up. *Gabrb3*^*ECKO*^ mice showed longer periods of immobility compared to sham control mice (Fig. 5b). Interneuron + PVEC co-transplanted *Gabrb3*^*ECKO*^ mice had a significantly lower immobility time, similar to sham controls. In contrast, interneuron-only *Gabrb3*^*ECKO*^ mice and interneuron + control endothelial cell co-transplanted *Gabrb3*^*ECKO*^ mice continued to show depressive-like behavior and had no significant improvement in immobility time (Fig. 5b). *Gabrb3*^*ECKO*^ mice showed high levels of anxiety that is assessed with the classic light–dark transition test. This test measures the animal’s innate preference for dark, enclosed places versus spontaneous exploratory behavior in bright, exposed places. Animals with increased anxiety spend less time in the light and more time in the dark part of the chamber. Interneuron-only *Gabrb3*^*ECKO*^ mice and interneurons + control endothelial cell co-transplanted *Gabrb3*^*ECKO*^ mice, similar to *Gabrb3*^*ECKO*^ mice, showed an aversion to light and preferred to remain in the dark. However, *Gabrb3*^*ECKO*^ mice co-transplanted with interneurons and PVECs spent equivalent times in light and dark areas, showing a rescue from increased anxiety-like behavior within 1 month of grafting (Fig. 5c). High levels of anxiety in *Gabrb3*^*ECKO*^ mice are also manifested by highly repetitive self-grooming behavior. Interneuron + PVEC co-transplanted *Gabrb3*^*ECKO*^ mice showed a significant reduction in self-grooming time compared to *Gabrb3*^*ECKO*^ mice, while interneuron-only and interneuron + control endothelial cells transplanted *Gabrb3*^*ECKO*^ mice continued to have a long self-grooming time (Fig. 5d). *Gabrb3*^*ECKO*^ mice have poor social communication skills. In a three-chamber social communication test, *Gabrb3*^*ECKO*^ mice showed no preference for a stranger mouse and spent approximately similar time in investigating stranger mouse versus an inanimate object. Like *Gabrb3*^*ECKO*^ mutants, interneuron-only *Gabrb3*^*ECKO*^ mice and interneuron + control endothelial cells co-transplanted *Gabrb3*^*ECKO*^ mice showed no preference for the stranger mouse (Fig. 5e). In contrast, interneuron + PVECs co-transplanted *Gabrb3*^*ECKO*^ mice interacted with the stranger mouse for a significantly longer duration than with the inanimate object (Fig. 5e; Supplementary Fig. 16). To confirm that the rescue of behavioral deficits in interneuron + PVEC co-transplanted *Gabrb3*^*ECKO*^ mice is due to the dispersion of GABAergic interneurons facilitated by PVECs, and not due to GABA released by PVECs only, we assessed behavioral function of PVECs-only transplanted *Gabrb3*^*ECKO*^ mice. Though, there was widespread distribution of human PVECs (Supplementary Fig. 17a–c), these mice continued to show behavioral deficits that were comparable to the *Gabrb3*^*ECKO*^ mice (Supplementary Fig. 17d–h). Together, these behavioral assays show that co-transplantation of PVECs and interneurons rescue behavioral deficits of *Gabrb3*^*ECKO*^ mice within 1 month of transplantation, as opposed to interneuron-only, PVEC-only or interneuron + control endothelial cell transplants that do not show a rescue effect during the same duration.

## Discussion

The results of our studies are notable for several reasons. First, they demonstrate the generation of specific human embryonic forebrain-like endothelial cells from human embryonic stem cells with emphasis on the importance of GABA and WNT7A addition for efficient differentiation, and isolation of the GABRB3^+^/CD31^+^ cell population by FACS (Fig. 5f). The general notion that it is not important which kind of human vasculature is used or that their purpose is to merely improve tissue survival and metabolic exchange should change. Endothelial cells from different organs are not exclusively homogenous, responding to the metabolic demands of cell populations. Cell autonomous programs within CNS endothelial cells dictate complex aspects of their vascular function and specific neurovascular interactions [12–15] that can be re-capitulated only by developing isogenic vasculature to induce precise structural organization. In that respect, human PVECs have significant potential as a source of cells for transplantation into the forebrain, and will have a broad impact on human brain development modeling and disease even beyond this specific research study. These cells will become invaluable for the induction of forebrain specific angiogenesis, aimed at the treatment of vascular diseases, cerebral ischemia or stroke, cortical lesions, and neurodegenerative disorders.

Second, our study illustrates the importance of our co-transplantation strategy with both endothelial and neuronal cell types and its significant benefits for brain repair. The identification of the molecular components involved in forebrain GABAergic interneuron development triggered the efficient generation of GABAergic neuronal populations based on ES cell engineering. However, these GABAergic interneurons have been used in transplantation without their natural substrate or guide, causing them to stall at transplantation sites with an inability to migrate into regions that require new neurons [23, 25, 33, 34]. Our results demonstrate how grafted human PVECs provide a migration promoting corridor that help human GABAergic interneurons migrate long distances within shorter periods of time to integrate themselves with the host tissue, thereby providing greater significance for faster repair of brain damage. Interestingly, when transplanted into the adult striatum, both endothelial cells and interneurons dispersed significantly and migrated both tangentially and long distance into the cerebral cortex, recapitulating the embryonic situation. This finding constitutes an important step toward the rational use of embryonic stem cell-derived PVECs in brain repair strategies targeting different regions of the forebrain (Fig. 5g).

Third, human PVECs may be of significance in an emerging field of brain organoid technology that is making remarkable progress. However, lack of blood vessels within growing brain organoids limits their application, both with respect to disease modeling and in the context of clinical transplantation. The current technique available for vascularization of organoids involves complex in vivo grafting of organoids [43]. Co-culture with PVECs is likely to improve forebrain organoid development, compartmentalization, and structure as well as minimize organoid to organoid growth and variability. Fourth, human PVECs can be generated from patient-derived iPSCs in diverse disease scenarios and may provide novel insights into disease etiology and pathogenesis.

Finally, our work emphasizes the importance of forebrain endothelial cell therapy for the improvement of behavioral function. The *Gabrb3*^*ECKO*^ mice have reductions in both blood vessels and GABAergic interneurons; therefore, transplantation of interneurons only, did not rescue the abnormal behavioral symptoms. Co-transplantation of both cell types was significant for the behavioral rescue. This illustrates the need for a greater understanding of both cell-type and region-specific defects with respect to psychiatric disorders for designing treatment strategies. It will be interesting in the future to extend this co-transplantation strategy to new disease models and to fine-tune the therapy with respect to the affected brain region, different neuronal cell types and long-term graft survival and safety. Thus, we expect our study to herald a new era in angiogenesis-specific treatment avenues for psychiatric disorders.

## Materials and methods

### Endothelial cell differentiation and culture

H9 cells (WiCell, Madison, WI) were maintained in E8 media (Thermo Fisher Scientific) on Matrigel (BD Biosciences)-coated plates and passaged once a week with 0.5 mM EDTA (Thermo Fisher Scientific) in PBS. On day 0 of differentiation, H9 cells were dissociated with Accutase (Sigma), and plated at a density of 10^5^ cells/cm^2^ on Matrigel-coated plates. Cells were cultured for 2 days in E8 medium supplemented with BMP4 (5 ng/ml, Peprotech), Activin (25 ng/ml, Peprotech), CHIR 99021 (1 µM,) WNT7A (500 ng/ml, Peprotech), and GABA (5 μM, Sigma). Rock Inhibitor, Y-27632 (10 μM, Selleck Chemicals) was added for first 24 h to improve cell survival. On day 2, cells were switched to vascular inducing medium composed of E6 medium (Thermo Fisher Scientific) containing BMP4 (50 ng/ml), SB431542 (5 μM, Cayman Chemicals), GABA (5 μM), WNT7A (500 ng/ml), FGF2 (100 ng/ml, Peprotech), and VEGF-A (50 ng/ml, Peprotech). On day 5, cells were split at 1:6 ratio using Accutase and plated on Matrigel-coated plates in PVEC medium consisting of E6 with VEGF-A (50 ng/ml), FGF2 (100 ng/ml), and GABA (5 μM). On day 7, PVECs were isolated from the mixed population by FACS and seeded on Matrigel-coated plates in PVECs medium at a density of 6 × 10^5^ cells/cm^2^. For routine culturing, PVECs were dissociated using Accutase, and seeded on Matrigel-coated plates at a density of 6 ×10^5^ cells/cm^2^ (high density seeding) or 1.2 × 10^5^cells/cm^2^ (low density seeding), with medium change every alternate day. PVECs were cryopreserved after at least one passaging in freezing medium composed of 90% PVECs medium and 10% DMSO. As a source of neurons, we used human iPSC-derived GABAergic neurons from Cellular Dynamics (Madison, WI, Cat# R1013). As control for microarray analysis and for cell migration assays, we used human-iPSC-derived endothelial cells from Cellular Dynamics (Madison WI, Cat# R1022), whose gene expression profile is similar to HAECs (https://fujifilmcdi.com/assets/CDI130925MIPTEC04.pdf). The human GABAergic neurons and human HAEC-like endothelial cells were cultured using the manufacturer’s protocol. Cell lines were routinely tested for mycoplasma contamination using a Mycoplasma Detection Kit (InvivoGen, San Diego, CA). Cells used in this study were verified to be mycoplasma free before undertaking any experiment with them.

### PVEC isolation by FACS

On day 7 of differentiation, cells were dissociated with Accutase and filtered through a 35 μm nylon mesh cell strainer cap to obtain a single cell suspension. Cell suspension was washed with ice-cold FACS buffer (2% FCS and 0.1% NaN_3_ in PBS) and incubated with Fcγ blocker (BD Biosciences Pharmingen, 1 µg/ml) for 30 min. Cells were then washed in ice-cold FACS buffer and stained with PE/Cy7 anti-human CD31 antibody (BioLegend, Cat # 303117) and anti-human GABRB3 antibody (Creative Diagnostics, Cat # DCABH-10376) conjugated with APC using an antibody conjugation kit (Abcam) for 1 h. Cells were washed in ice-cold FACS buffer and CD31^+^GABRB3^+^ endothelial cells were isolated by FACS using a BD FACS Aria-II flow cytometer (BD Biosciences, San Jose, CA).

### Gene expression profile analysis

Total RNA was extracted using the RNeasy plus minikit (Qiagen) according to the manufacturer’s instruction. RNA quality was determined using nanodrop and an Agilent 2100 bioanalyzer. Microarray hybridization was performed using the Human Gene Array 2.0 ST gene chip (Affymetrix) at the Boston University Microarray and Sequencing Resource Core, Boston, MA. Principal component analysis (PCA) was performed after normalizing gene-level expression values from CEL files of Affymetrix human gene 2.0 ST arrays by using the implementation of the Robust Multiarray Average in the Affymetrix transcriptome analysis console (TAC) (v4.0.1, Applied Biosystem, Foster City, CA, USA). For exploratory group analysis, a Volcano plot, and a hierarchical clustering heatmap using TAC software were created after curating with a threshold parameter, 1.5-fold expression, *P* < 0.05, FDRq < 0.1. Relative Log Expression and Normalized Unscaled Standard Error using the affyPLM package (version 1.34.0) and differential expression were assessed using the moderated (empirical Bayesian) *t* test implemented in the limma package (v 3.14.4) [44]. The Heatmap visualization was performed using Morpheus (Broad Institute, Boston, MA, USA). Violin plot visualization for expression level comparison of samples was generated with the log2 expression value using GraphPad Prism v8.0 (GraphPad Software, La Jolla, CA, USA). The gene ontology for gene enrichment study was performed in three GO TERM annotation categories by using the Database for Annotation, Visualization, and Integrated Discovery v6.8 with the modified Fisher’s exact test [45] and was visualized using GraphPad Prism software.

#### Tube-formation assay

For the Matrigel-based tube-formation assay, 10^5^ PVECs (at passage number P2 or P3) were suspended in 400 µl of PVECs medium and seeded in one well of 24-well culture plates precoated with growth factor-reduced Matrigel (BD Biosciences). Cells formed tubular structures within 24 h, which were imaged using a light microscope. The ability of PVECs to form tubes in 3D was assessed using the Fibrin Gel In Vitro Angiogenesis Assay Kit (Chemicon). A total of 5 × 10^5^ cells/ml of medium were seeded in one well of a 24-well plate coated with a fibrin matrix as described by the manufacturer. After 24 h, cells were covered with a second layer of fibrin and fresh medium added. Capillary tube networks formed inside the fibrin gel within 2 days and were imaged using a light microscope.

#### Sprouting assay

Sprouting of PVECs was observed using a fibrin gel bead assay. Briefly, 10^6^ PVECs (at passage P2 or P3) were coated onto 2500 Cytodex beads and allowed to attach overnight. Next day, beads were embedded in fibrin gels in 24-well culture plates (500 beads/ml), and human primary lung fibroblasts (ATCC) were plated on top of the gel (20,000 fibroblasts/well). Budding and sprouting of endothelial cells from the beads were observed from day 2 onward and lumen formation was visible from day 4.

#### Long-distance migration assay

In preparation for migration assays, two-well silicone culture inserts (from ibidi GmbH, Cat# 80209) were converted into one-well inserts by cutting with a sharp, sterile blade. Individual one-well inserts were placed in the middle of a 35-mm dish coated with poly-ornithine and laminin. The boundary of the insert was marked on the back of each dish with a thin marker. For this assay, PVECs, control endothelial cells and endothelial cells derived without GABA and WNT7A were used at passage number P2 or P3, while GABA interneurons cultured for 6 weeks were used. In total, 10^4^ PVECs or endothelial cells derived without GABA and WNT7A were seeded in each insert in PVEC medium without GABA. Same number of GABAergic interneurons or control endothelial cells were seeded per insert in their respective manufacturer’s recommended medium. The insert was removed after 48 h and cells cultured for 5 days. After 5 days, cells were fixed and fluorescently labeled with an anti-human CD31 antibody (for endothelial cells) or an anti-human β-tubulin antibody (for neurons) and DAPI. The distance between each cell body and the edge of the insert boundary was measured using ImageJ.

#### Co-culture migration assay

PVECs or control endothelial cells at passage number P2 or P3, and GABA interneurons cultured for 6 weeks were used for this assay. A total of 3 × 10^4^ GABAergic interneurons and 3 × 10^4^ PVECs were co-suspended in 70 µl of co-culture medium (50% PVEC medium without GABA and 50% GABA neuron maintenance medium from Cellular Dynamics) and seeded in a one-well insert (prepared as described above) in a poly-Ornithine/Laminin coated 35-mm dish. As control, 3 × 10^4^ GABAergic interneurons only or 3 × 10^4^ GABAergic interneurons and the same number of control endothelial cells were co-seeded. Inserts were removed after 2 days, and co-culture was maintained for 5 days. After 5 days, cells were fixed and double labeled with anti-human CD31 and anti-human β-tubulin antibodies. Neuronal migration was assessed by measuring the distance traveled by β-tubulin^+^ neurons from the day 0 mark using ImageJ software.

#### Chemoattractivity assay

For this assay PVECs at passage number P2 or P3, control endothelial cells at passage P2, and GABA interneurons cultured for 6 weeks were used. Three-well culture inserts (ibidi GmbH, Cat # 80369) were placed in the center of poly-Ornithine/laminin coated 35 mm dish. A total of 3 × 10^4^ GABA neurons were seeded in the center well. Equal number (10^4^ cells) of PVECs and control endothelial cells were seeded in the two-side wells. The inner edge of the neuronal well was demarcated at the back of the dish using a thin sharpie. Inserts were removed one day post-seeding, and cells were fixed after 36 h. Cells were double labeled with anti-human CD31 and anti-human β-tubulin antibodies and imaged. The chemo-attractive response of β-tubulin^+^ neurons toward endothelial cells in each experiment were imaged and quantified using a scoring scheme modified from Won et al. [13].

#### Migration assays with chemicals

To assess the roles of GABA or SDF-1/CXCL12 signaling on migration of human PVECs, 10^4^ cells were seeded in one-well insert and allowed to migrate in the presence of respective agonist or antagonist in PVEC medium (without GABA). After 5 days, cells were fixed, stained with anti-human CD31 antibody (Millipore), imaged and the distance migrated was calculated using ImageJ. To examine the effect of endothelial GABA or endothelial SDF-1/CXCL12 signaling on interneuron migration, human PVECs were seeded in 35-mm dish (10^5^ cells/cm^2^) and incubated with respective chemical for a period of 48 h. A total of 3 × 10^4^ GABAergic interneurons were seeded on top of the PVECs using a one-well insert, and allowed to migrate over the pre-incubated PVECs for 2 days. Migration of neurons was assayed by staining with anti-human β-tubulin antibody (Biolegend) and imaged. The concentration of chemicals used in the assays are as follows: muscimol (Sigma) 100 µM, BMI (Sigma) 100 µM, AMD3100 (Sigma) 50 µM, recombinant human SDF-1α (Peprotech) 40 nM. The chemicals were kept at −20° as concentrated stock solutions and diluted on the day of the experiment.

### Animals

Adult NOD-SCID mice (8 weeks old) were purchased from Charles River Laboratories, MA. *Tie2-cre* mice and *Gabrb3 floxed* (*Gabrb3*^*fl/fl*^) mice were obtained from Jackson Labs. The *Tie2-cre* transgene is known for uniform expression of cre-recombinase in endothelial cells during embryogenesis and adulthood [14, 15]. To selectively delete *Gabrb3* in endothelial cells, *Tie2-cre* transgenic mice (males) were crossed to *Gabrb3*^*fl/fl*^ mice (females) to generate *Tie2-cre; Gabrb3*^*fl*/+^ mice (males). These were further crossed with *Gabrb3*^*fl/fl*^ mice (females) to obtain the *Gabrb3* conditional knockout (*Tie2-cre; Gabrb3*^*fl/fl*^ mice). Animal experiments were in full compliance with the NIH Guide for Care and Use of Laboratory Animals and were approved by the HMRI and McLean Institutional Animal Care Committees (IACUC).

### Stereotaxic surgery and cell transplantation

NOD-SCID mice, *Gabrb3*^*fl/fl*^ mice and *Gabrb3*^*ECKO*^ mice were housed on a 12 h light/dark cycle and had free access to food and water throughout the study. Eight-week-old mice were used for all transplantations. Cells for transplantation were suspended in transplantation medium composed of DMEM/F-12 with no phenol red (Thermo Fisher Scientific), BDNF (10 ng/ml, Peprotech), GDNF (10 ng/ml, Peprotech), Rock-Inhibitor (10 uM), and Boc-Asp(OMe) fluoromethyl ketone (20-µM, Cayman Chemicals). For co-transplantation, PVECs or control endothelial cells derived without WNT7A and GABA (at passage P2 or P3; 50,000 cells/µl) and GABAergic interneurons (at 6 weeks of differentiation; 50,000 cells/µl) were suspended in a 1:1 ratio. For interneuron-only or endothelial cell-only transplants, cells were suspended at a concentration of 50,000 cells/µl. Before surgery, mice were anesthetized with 4% isoflurane, and kept under 2% isoflurane gas throughout the procedure. All microinjections were performed through a pulled borosilicate glass pipette with a long, gently tapering shank using an UMP microsyringe pump (World Precision Instruments) and a Kopf stereotaxic frame (Kopf Instruments, CA). One microliter of cell solution was injected at a rate of 0.125 μl/min. After each injection, the microcannula remained in position for 5 min before withdrawing slowly to avoid back-flow of cells. Injection coordinates were as follows: for striatum–bregma: 0.49 mm, ventral: −3.0 mm, lateral: −1.8 mm; for neocortex-bregma: 0.49 mm, ventral: 1.8 mm, lateral: 2.0 mm. Transplanted *Gabrb3*^*ECKO*^ mice received subcutaneous injections of cyclosporine (35 µl of 50 mg/ml stock, Perrigo), beginning two days before surgery, and continuing every day until mice were sacrificed. Transplanted mice were terminally anesthetized with a Ketamine/Xylazine cocktail (100 and 10 mg/kg, respectively) and perfused intracardially with cold 4% formaldehyde for cryo-processing and IHC, or with a zinc fixative (BD Biosciences Pharmingen) for paraffin histology and IHC.

#### Immunohistochemistry (IHC)

For frozen section IHC, PFA-perfused brains were post-fixed in cold 4% formaldehyde for 48 h, cryo-protected in a sucrose gradient, flash frozen in dry ice, and cryo-sectioned into 40 µm coronal sections. For immunostaining, sections were washed once with PBS, blocked in FBS containing 0.5% Triton X100 for 1 h, and incubated with the primary antibody overnight at 4 °C. The following day, slides were washed six times with PBS at room temperature, incubated with secondary antibodies (Alexa-568 and Alexa-488, 1:400) for 2 h at room temperature, washed with PBS six times, and mounted onto slides with a DAPI-containing mounting medium (Vectashield). For paraffin IHC, brains were post fixed in Zinc fixative (BD Biosciences Pharmingen) for 48 h, dehydrated in an alcohol gradient (70, 80, 95, 100%), cleared in Xylene, embedded in paraffin wax, and sectioned into 8-µm coronal sections. Prior to immunostaining of paraffin sections, tissue was deparaffinized and antigen retrieval was performed in a pH 9 solution (DAKO) at 96 °C. Primary antibodies used for IHC were as follows: anti-human CD31 (1:100, Biolegend), anti-human vWF (1:100, Sigma), anti-human nuclei (1:100, Rockland), anti-human mitochondria (1:100, Millipore), anti-human β-TUBULIN (1:2000, Biolegend), anti-GABA (1:1000, Sigma), anti-GABRB3 (1:200, Sigma), anti-Caspase (1:50, Millipore), anti-Claudin 5 (1:200, Sigma), anti-human Ki67 (1:50, Thermo Fisher Scientific), anti- ZO-1 (1:400, Thermo Fisher Scientific), isolectin B4 (1:50, Sigma), anti-NKX2.1 (1:250, Abcam), anti-OCT4 (1:200, SCBT), ant-TRA1-60 (1:200, Millipore). The secondary antibodies used were Alexa-594 and Alexa-488 (1:400, Thermo Fisher Scientific).

#### Immunocytochemistry

Cells were grown to 70–80% confluency on coverslips, fixed in 4% PFA for 15 min at room temperature, blocked in blocking solution (PBS supplemented with 1% Bovine Serum and 0.25% Triton X) for 1 h at room temperature, and incubated with the primary antibodies overnight in 4 °C. Next day, cells were stained with a secondary antibody (Alexa-594 or Alexa-488, 1:500, Thermo Fisher Scientific) for 2 h at room temperature and mounted using ProLong™ Diamond Antifade Mountant with DAPI (Thermo Fisher Scientific). The primary antibodies were same as that for IHC and used at same dilutions (mentioned above), except for anti-human CD31 which was purchased from Millipore and used at a dilution of 1:100.

#### H and E staining

Brains were post fixed in a Zinc fixative (BD Biosciences Pharmingen) for 48 h, dehydrated in an alcohol gradient (70, 80, 95, 100%), cleared in Xylene, embedded in paraffin wax, and sectioned into 8 µm coronal sections. Briefly, slides were deparaffinized in Xylene, hydrated in ethanol gradient (100, 95, 70%), rinsed in tap water, stained with Hematoxylin for 1 min, rinsed again in tap water, stained in Eosin for 30 s, dehydrated in an ethanol series (70, 95, 100%) followed by xylene, and mounted onto glass slides in permount (Sigma).

#### Microscopic analysis and cell counting

Twenty sections from each brain were used for IHC and histology experiments. All low and high magnification images were obtained with a FSX100 microscope (Olympus). Counting of human nuclei^+^ cells in each type of transplant was performed using ImageJ. The number of transplanted neurons that had migrated into the cerebral cortex was obtained by counting human nuclei^+^ and β-tubulin^+^ cells using the ImageJ software. Blinding was performed during cell counting analysis.

### Behavioral experiments

All behavioral tests were done during the light phase of the light/dark cycle. Before behavioral testing, mice were acclimatized to the testing room for 1 h. Behavioral assays were performed according to established protocols referenced here: nest building with shredded paper [46], self-grooming [47], light–dark box [48], tail suspension test [49], and three-chamber social interaction test [50]. Both males and females were used for all behavioral assays. Experimenters scoring behaviors were blinded to the genotypes. Results were analyzed using Student’s *t* test, and one-way ANOVA. Data are presented as mean ± standard deviation. Values of *p* < 0.05 were considered statistically significant.

### ELISA

ELISA was used to detect and compare GABA levels released from human interneurons only, human PVECs-only and PVECs-neuron co-culture. PVECs-only and interneuron-only cultures were prepared by seeding cells in a 12-well plate at a density of 10^5^ cells/cm^2^. The endothelial and interneuron co-culture was prepared by co-seeding both cells at a 1:1 ratio with a final density of 10^5^ cells/cm^2^. Supernatants from cells were collected after 96 h and stored at −80 °C. GABA concentrations were quantitatively determined by competitive ELISA according to the manufacturers’ protocol (GABA Research ELISA kits, Labor Diagnostica Nord, Germany), and absorbance was measured using a multiplate microplate fluorescence reader (Molecular Devices, CA) at 450 nm.

### Quantitative real-time PCR

Total RNA was prepared using the RNeasy plus minikit (Qiagen). cDNA from total RNA was generated using the SuperScript™ III First-Strand Synthesis System (Thermo Fisher Scientific) as per manufacturer’s protocol. PCR reactions were run on a CFX96 Touch Real-Time PCR (Bio-Rad) with SsoAdvanced™ Universal SYBR^®^ Green Supermix (Bio-Rad). Primers for qPCR were obtained from Thermo Fisher Scientific. The relative expression level for each gene was normalized to that of GAPDH gene and subsequent fold changes were determined according to published methodology [51].

### Western blot

Cell lysates were prepared in standard radioimmunoprecipitation buffer containing 1× protease Inhibitor cocktails, and protein was quantified using Bio-Rad protein quantification assay. Cell lysates were then separated in a 12% gel, and immunoblotted onto a nitrocellulose membrane using the NOVEX SDS electrophoresis unit. Membrane was blocked for 1 h at RT using the Odyssey blocking buffer (Licor), and incubated with primary antibodies; anti-CD31 (Thermo Fisher) and anti-VE-cadherin (Sigma) and anti-GAPDH (Proteintech) overnight at 4 °C. Next day, membranes were washed and incubated with secondary antibodies, anti-mouse IRD 800 and anti-rabbit IRD 680 for 1 h at RT in dark. Fluorescent signal was detected using Licor detection system and data analyzed using the lmageLite studio software.

### Statistical analysis

All statistical analyses were performed using GraphPad Prism 7 (GraphPad Software, La Jolla CA). Power analysis was performed using Java Applets for Power and Sample size. Simple random sampling was used. Sample sizes assigned to the same experimental groups were equal. The exact sample size (*n*) for each experimental group is reported in individual figure legends. For in vitro assays, the number of cells examined, is mentioned in respective methods sections. For histology and IHC experiments, 20 sections from each brain were used for each experimental group. Uniform penetration of antibodies or stains throughout the section was ascertained and quality of the staining in each digital section was examined. Only those sections which showed uniform labeling were included in further analysis. Blinding was used during cell counting analysis and behavioral experiments. No data were excluded. Statistical significance of differences between groups was analyzed by two-tailed Student’s *t* test (Prism; GraphPad software) and has been noted in individual figure legends. Significance was reported at *p* < 0.05.

## Supplementary information

Supplementary Material
