# Supplementary material for: Human forebrain endothelial cell therapy for psychiatric disorders
Source: Mol Psychiatry. 2020 Jul 13;26(9):4864–83. doi: 10.1038/s41380-020-0839-9 (PMC8162704; doi:10.1038/s41380-020-0839-9)
Supplement: Supplementary file 1 — Supplementary Material [file 41380_2020_839_MOESM1_ESM.pdf]

Supplementary Information

Human Forebrain Endothelial Cell Therapy for Psychiatric Disorders

Debkanya Datta[1,2,3], Sivan Subburaju[1,2,3], Sarah Kaye[1,3], Jugajyoti Baruah[1,2,3], Yong Kee Choi[1,2,3], Yeqi Nian[4,5], Jahan S Khalili[6], Sangmi Chung[7], Abdallah Elkhal[4,5] and Anju Vasudevan[1,2,3 ϕ]

[1] Angiogenesis and Brain Development Laboratory, Huntington Medical Research Institutes (HMRI), 686 S Fair Oaks Avenue, Pasadena, CA, 91105, USA.
[2] Department of Psychiatry, Harvard Medical School, Boston, MA, 02215, USA.
[3] Division of Basic Neuroscience, McLean Hospital, 115 Mill Street, Belmont, MA, 02478, USA.
[4] Department of Surgery, Harvard Medical School, Boston, MA, 02115, USA.
[5] Division of Transplantation, Brigham and Women's Hospital, 221 Longwood Avenue, EBRC 309, Boston, MA, 02115, USA.
[6] Personal Peptides LLC, Houston, Texas, 77002, USA.
[7] Department of Cell biology and Anatomy, New York Medical College, Valhalla, NY 10595, USA.

[ϕ] Correspondence should be addressed to: A.V. (Anju.Vasudevan@hmri.org)

Supplementary Figure 1:

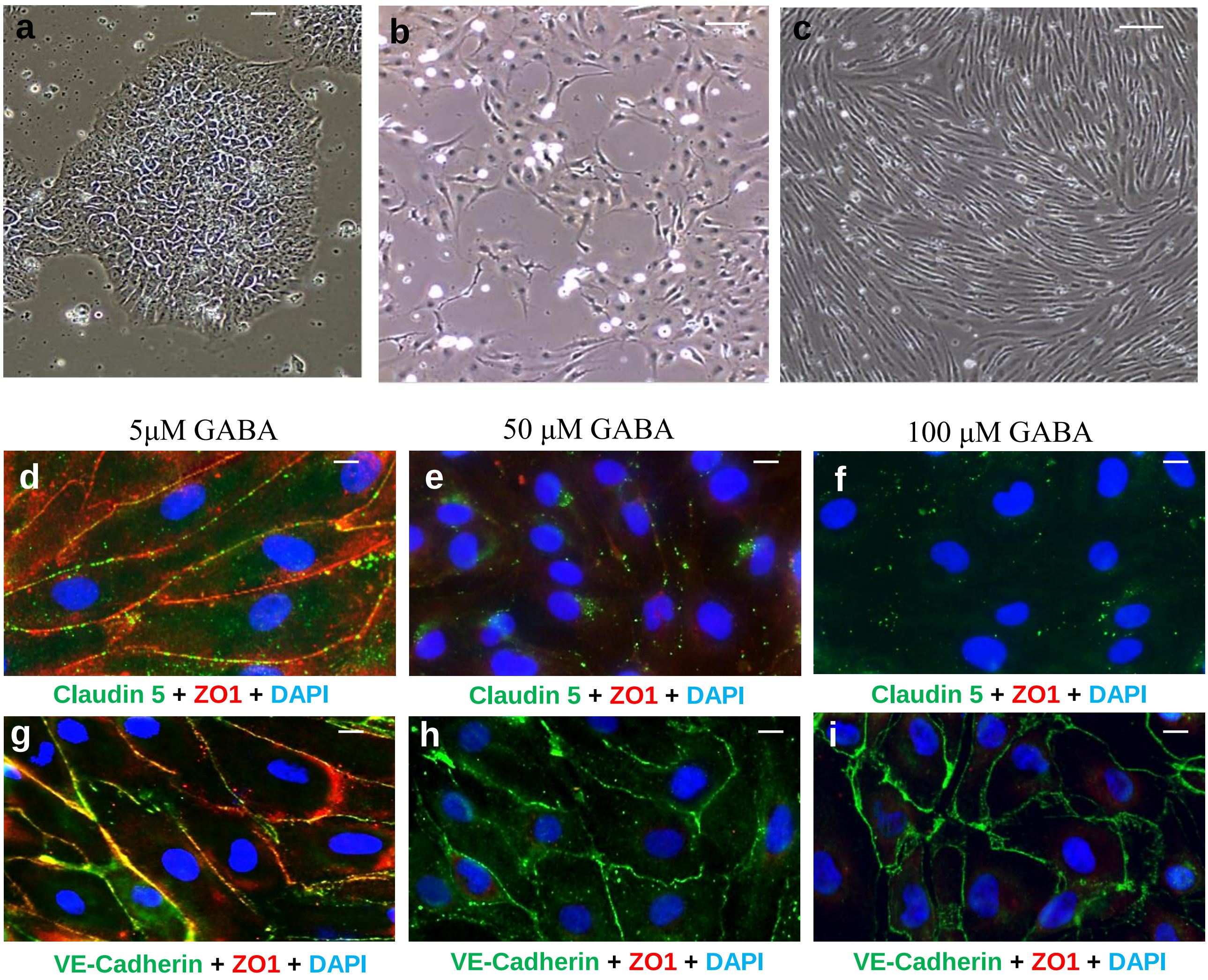

Supplementary Figure 1: **Human periventricular endothelial cell morphology and effect of GABA**. Phase contrast images of: (a) an undifferentiated colony of the embryonic stem cell line H9, (b) human periventricular endothelial cells after 2 days of splitting and (c) a confluent culture of human periventricular endothelial cells. (d-i) GABA concentration impacts tight junction formation in human periventricular endothelial cells. (d-f) Addition of 5uM GABA increases tight junction formation (d), observed by antibody staining against tight junction proteins Claudin5 and ZO-1, in comparison to higher concentration of 50μM (e) or 100μM (f) GABA. (g-i) Expression of VE-Cadherin, an adherens junction protein, is not affected by increase in GABA levels. Scale bar: a, 50μm (applies to d-i); b, 100 um (applies to c).

Supplementary Figure 2:

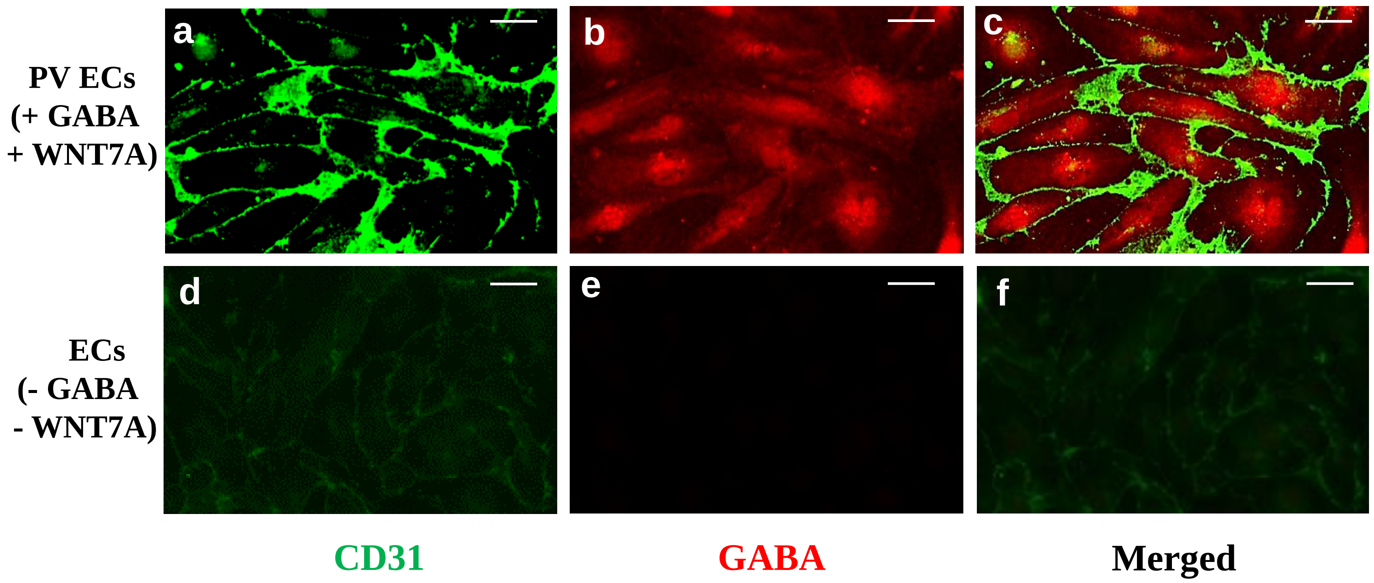

Supplementary Figure 2: Effect of GABA and WNT7A. Double staining with antibodies against CD31 (green) and GABA (red) show that GABA and WNT7A are required for the expression of endogenous GABA in human periventricular endothelial cells. (a-c) Periventricular endothelial cells express GABA when derived in the presence of GABA and WNT7A. (d-f) GABA is not detected in endothelial cells derived without GABA and WNT7A. These cells also have significantly lower levels of CD31 (shown also in Supplementary Figure 3c and Supplementary Figure 4). Scale bar: a, 100 μm (applies to b-f).

Supplementary Figure 3:

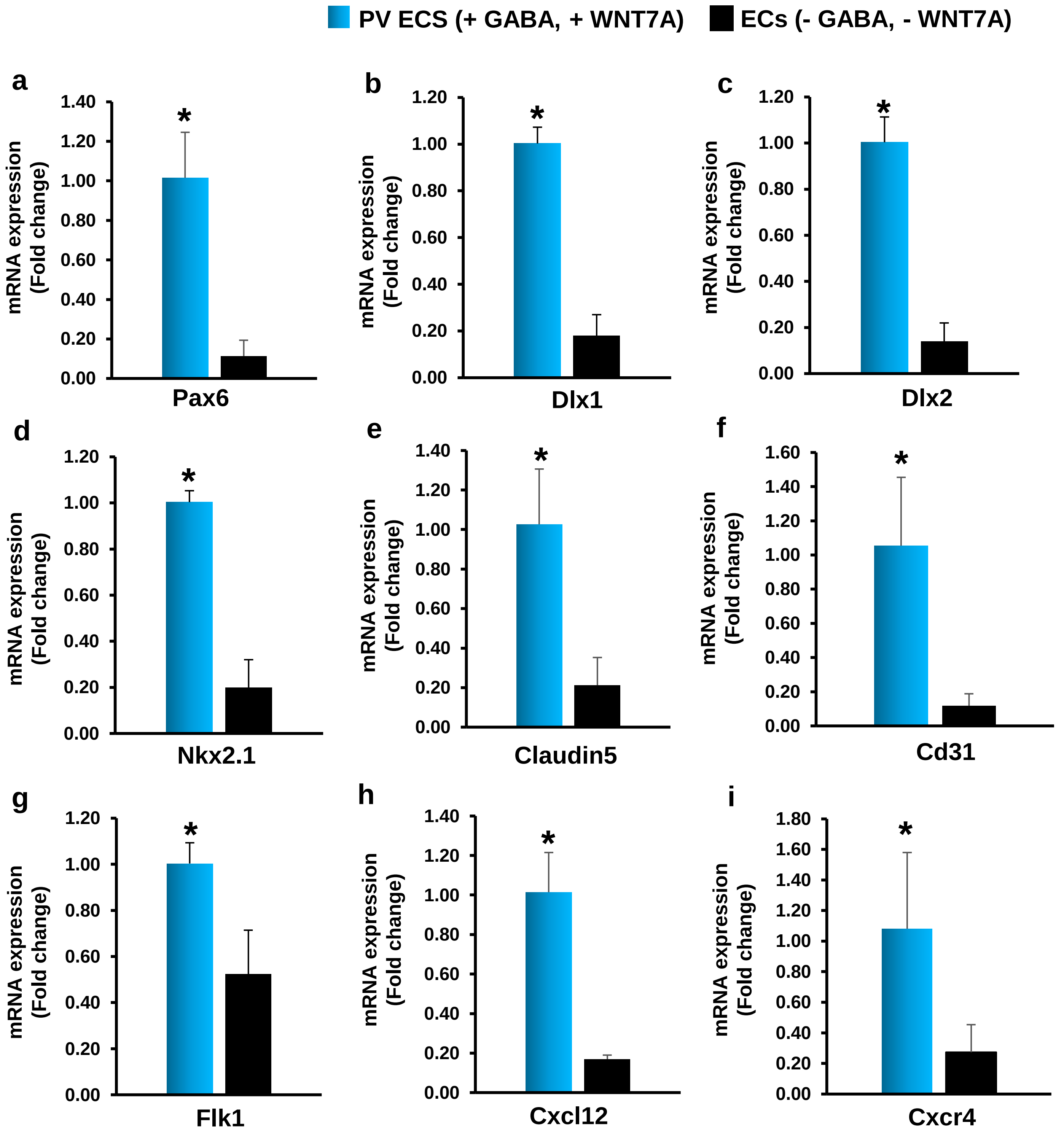

Supplementary Figure 3. **Differences in gene expression between human periventricular endothelial cells and control endothelial cells derived without GABA and WNT7A.** Real time qPCR analysis show decreased levels of *Pax6*, *Dlx1*, *Dlx2*, *Nkx2.1*, *Claudin 5*, *Cd31*, *Flk1*, *Cxcr4* and *Cxcl12* in control endothelial cells derived without GABA and WNT7A, in comparison to human periventricular endothelial cells. Bar graphs show fold change ± SD values of relative transcript expression normalized to GAPDH and compared with values obtained from three independent experiments (n=3, * $P < 0.05$, Student's t test).

Supplementary Figure 4:

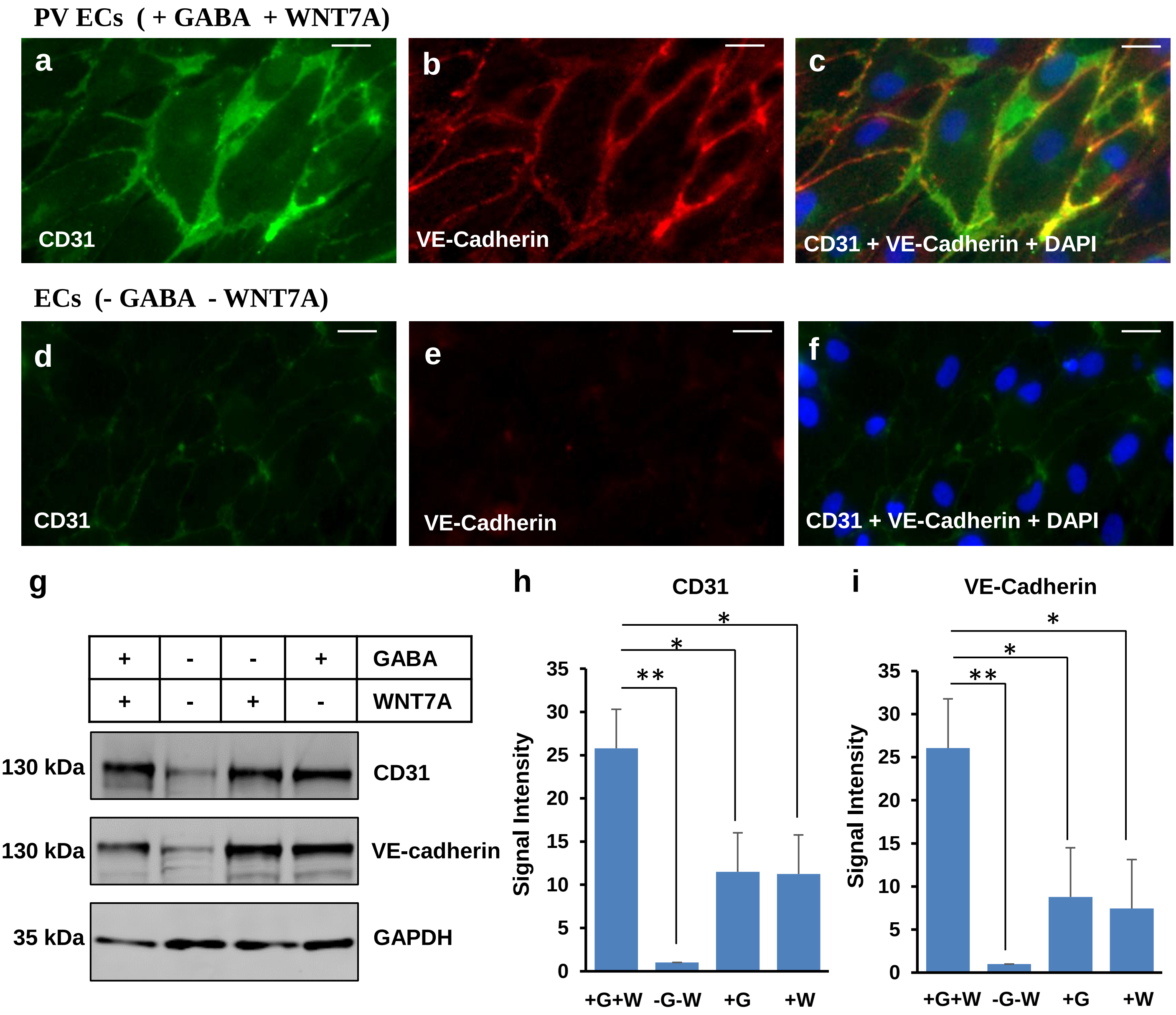

Supplementary Figure 4: Decrease in expression of CD31 and VE-Cadherin in endothelial cells derived without GABA and WNT7A. (a-f) Immunocytochemical staining show substantially low expression of CD31 and VE-Cadherin in endothelial cells that are generated without addition of GABA and WNT7A (d-f), in comparison to human periventricular endothelial cells (a-c). (g) A representative western blot image showing CD31 and VE-Cadherin protein levels in human periventricular endothelial cells (+GABA, +WNT7A) versus in endothelial cells derived without GABA and WNT7A, with WNT7A but not GABA, and with GABA but not WNT7A. (h,i) Quantification of signal intensities from western blot confirms significant decrease in levels of CD31 and VE-Cadherin proteins in endothelial cells when derived without addition of GABA and/or WNT7A. Each data point represents mean ± SD values calculated from three independent biological replicates per group. (n=3, * $P<0.05$, ** $P<0.01$, Student's t test). G: GABA; W: WNT7A; PV ECs: periventricular endothelial cells

Supplementary Figure 5:

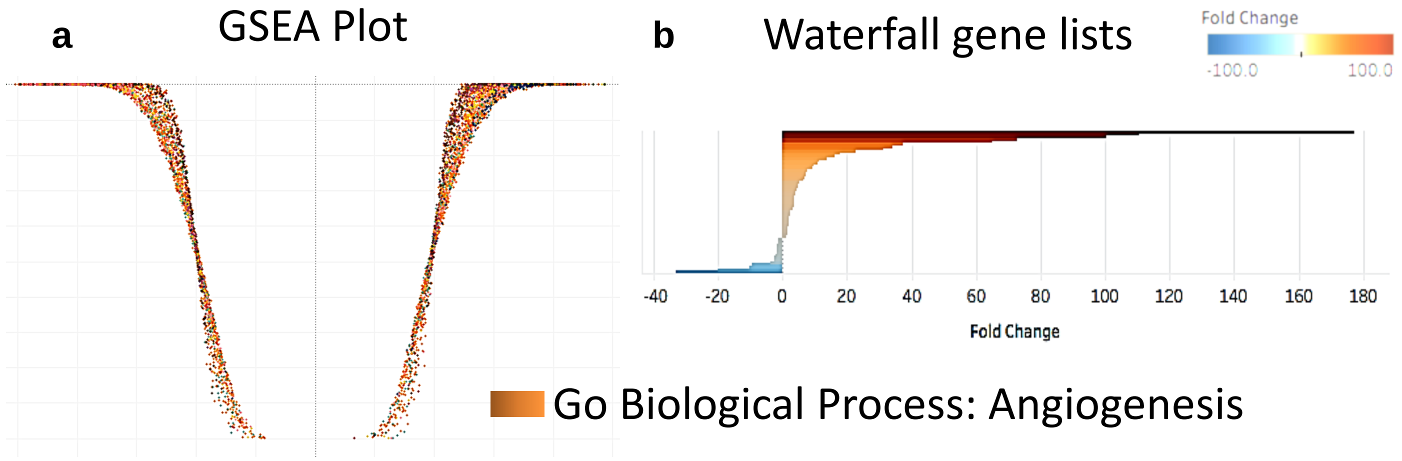

c

Group	Gene Set Name	Enrichment Score (ES)	Normalized Enrichment Score (NES)	Gene Set Size
GO Biological Process	GO_ANGIOGENESIS	0.6	2.4	289.0
	GO_REGULATION_OF_ENDOTHELIAL_CELL_MIGRATION	0.7	2.3	111.0
	GO_NEGATIVE_REGULATION_OF_BLOOD_VESSEL_ENDOTHELIAL_CELL_MIGRATION	0.8	2.2	23.0
	GO_ENDOTHELIUM_DEVELOPMENT	0.6	2.2	89.0
	GO_REGULATION_OF_BLOOD_VESSEL_ENDOTHELIAL_CELL_MIGRATION	0.7	2.2	50.0
	GO_NEGATIVE_REGULATION_OF_ENDOTHELIAL_CELL_MIGRATION	0.7	2.2	38.0
	GO_POSITIVE_REGULATION_OF_ENDOTHELIAL_CELL_MIGRATION	0.7	2.2	66.0
	GO_REGULATION_OF_ENDOTHELIAL_CELL_DIFFERENTIATION	0.8	2.1	27.0
	GO_VASCULAR_ENDOTHELIAL_GROWTH_FACTOR_RECEPTOR_SIGNALING_PATHWAY	0.6	2.1	71.0
	GO_ENDOTHELIAL_CELL_MIGRATION	0.7	2.1	54.0
	GO_REGULATION_OF_CELL_MIGRATION_INVOLVED_IN_SPROUTING_ANGIOGENESIS	0.8	2.0	19.0
	GO_REGULATION_OF_SPROUTING_ANGIOGENESIS	0.7	2.0	27.0
	GO_CELLULAR_RESPONSE_TO_VASCULAR_ENDOTHELIAL_GROWTH_FACTOR_STIMULUS	0.7	2.0	30.0
	GO_REGULATION_OF_VASCULAR_ENDOTHELIAL_GROWTH_FACTOR_RECEPTOR_SIGNALIN..	0.7	2.0	26.0
	GO_ENDOTHELIAL_CELL_PROLIFERATION	0.8	2.0	22.0
	GO_REGULATION_OF_ENDOTHELIAL_CELL_APOPTOTIC_PROCESS	0.6	2.0	42.0
	GO_SPROUTING_ANGIOGENESIS	0.6	2.0	44.0
	GO_ENDOTHELIAL_CELL_DIFFERENTIATION	0.6	1.9	71.0
	GO_NEGATIVE_REGULATION_OF_ENDOTHELIAL_CELL_PROLIFERATION	0.7	1.9	32.0
	GO_POSITIVE_REGULATION_OF_ENDOTHELIAL_CELL_DIFFERENTIATION	0.8	1.9	15.0
	GO_REGULATION_OF_SYSTEMIC_ARTERIAL_BLOOD_PRESSURE_BY_RENIN_ANGIOTENSIN	0.7	1.9	22.0
	GO_MORPHOGENESIS_OF_AN_ENDOTHELIUM	0.8	1.9	16.0
	GO_REGULATION_OF_ENDOTHELIAL_CELL_PROLIFERATION	0.5	1.9	97.0

Supplementary Figure 5: **Gene expression profile in human periventricular endothelial cells and H9.** (a) GSEA plot shows an enrichment of Go Biological Process (orange): Angiogenesis in human periventricular endothelial cells versus H9 undifferentiated cells. (b) The waterfall gene list shows a significant increase in angiogenesis categories (orange) in human periventricular endothelial cells. (c) The enrichment in angiogenesis/endothelial gene set categories in human periventricular endothelial cells is further elaborated in the table.

Supplementary Figure 6:

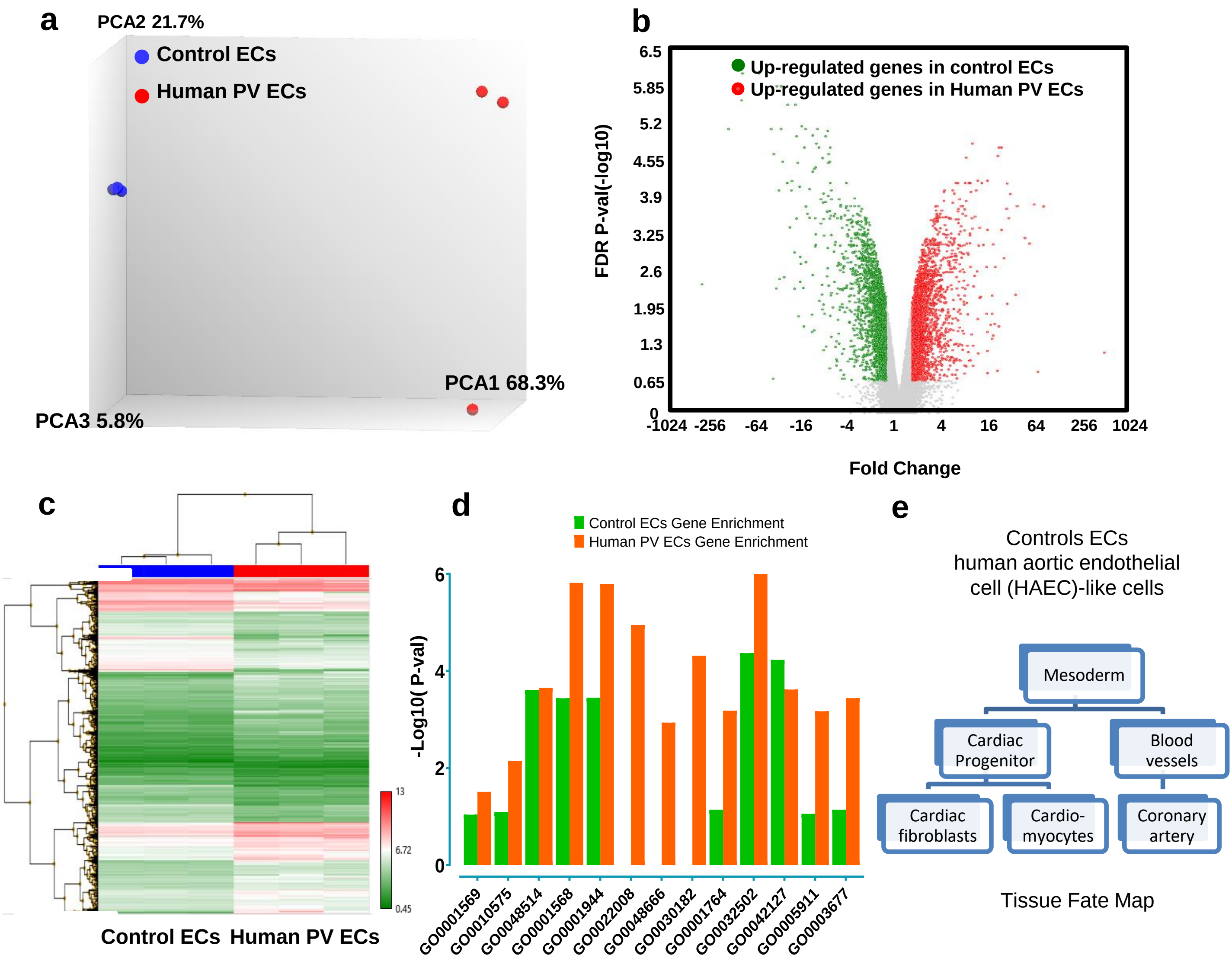

Supplementary Figure 6: Gene expression profile in human periventricular endothelial cells and control endothelial cells. (a) Principal component analysis (PCA) of microarrays. PCA plot of three control endothelial cells (ECs) samples, (Blue) and three human periventricular (PV) ECs (Red) samples showed clear separation along the PCA1 axis. (b) Volcano plot shows differentially expressed genes between control ECs (Green) and human PV ECs (Red). 3820 genes were selected as significantly different and were sorted by P value (<0.05) and fold change (>1.5). (c) Hierarchical clustering of selected genes of control ECs (Blue) and human PV ECs (Red) shows differentially expressed genes. Cell samples are in columns and genes are in rows. (d) Comparison of gene enrichment between control ECs (Green) and human PV ECs (Orange) in specific Gene Ontology (GO) categories of biological process (BP) are represented as –log10 (P value) sorted by P<0.05 in the bar graph. GO categories in this analysis include: GO0001569 (Patterning of blood vessels), GO0010575 (Positive regulation of vascular endothelial cell growth factor production), GO0048514 (Blood vessel morphogenesis), GO0001568 (Blood vessel development), GO0001944 (Vasculature development), GO0022008 (Neurogenesis), GO0048666 (Neuron development), GO0030182 (Neuron differentiation), GO0001764 (Neuron migration), GO0032502 (Developmental process), GO0042127 (Regulation of cell proliferation), GO0005911 (Cell-cell junction) and GO0003677 (DNA binding). (e) Tissue fate mapping of control endothelial cell gene expression confirmed their cardiac-like identity.

Supplementary Figure 7:

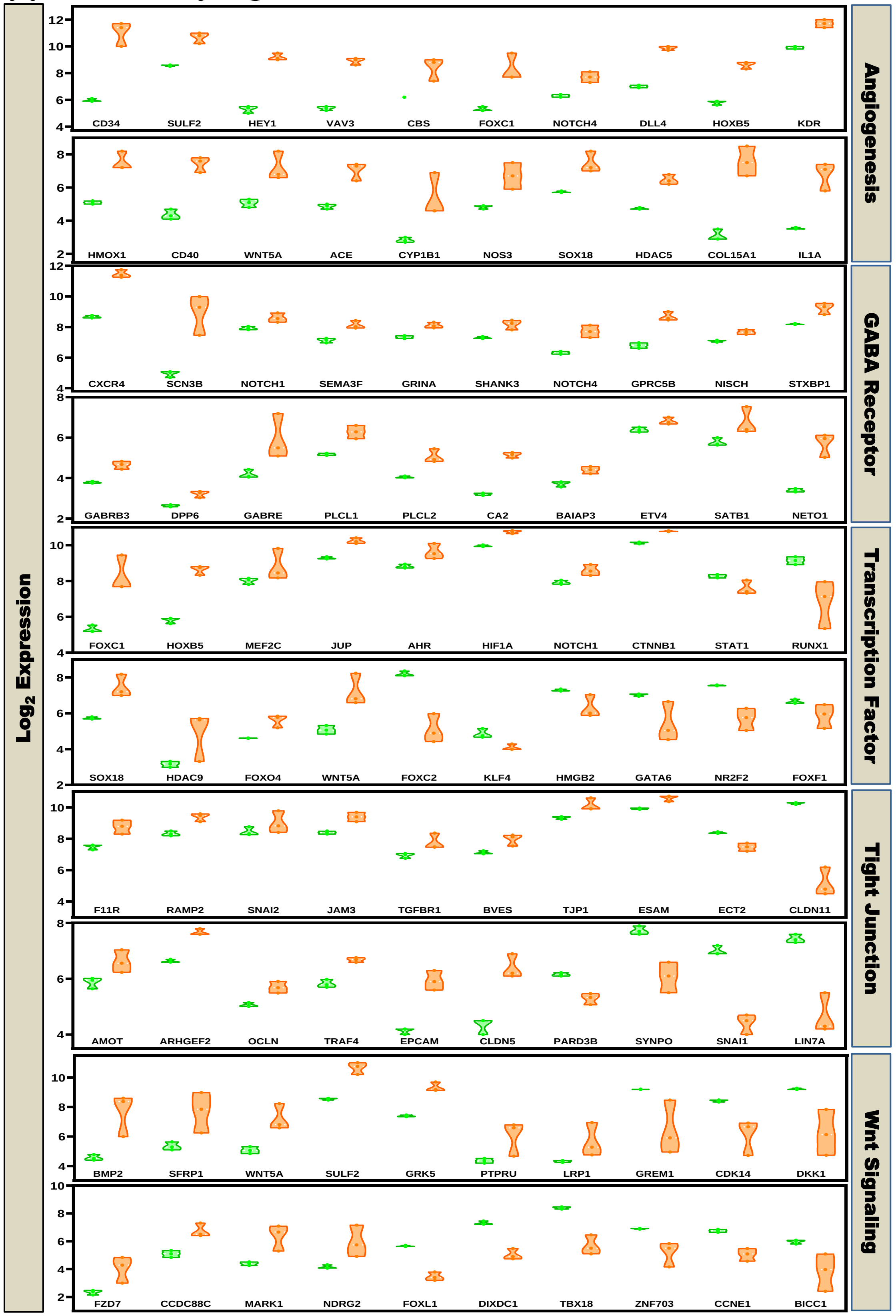

Supplementary Figure 7: Differentially expressed gene categories in human periventricular endothelial cells (PV ECs) versus control endothelial cells (ECs). Violin plot shows 5 specific categories of genes that are up- or down-regulated significantly in human PV ECs (orange) versus control ECs (green). Expression levels in Y-axis are represented as binary logarithm (log2) value. Each category is indicated on the right side. Differential expression of each gene between control ECs and human PV ECs is $P<0.05$.

Supplementary Figure 8:

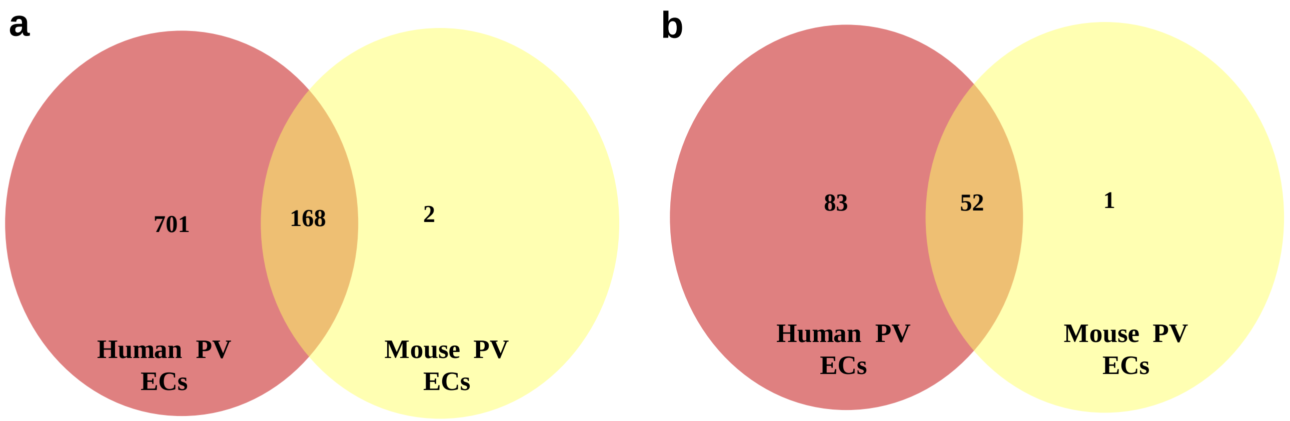

Table 1

MMP2, COL3A1, CADM4, CSPG4, FLT1, ALOX12, TBX4, KRIT1, RORA, ANGPTL4, LEP, GPNMB, FOXC2, TGM2, ADCYAP1, NTRK2, NOS1, SMO, COL1A1, SLC8A1, ISL1, PRKG1, CLEC14A, ADRA2B, PAX6, SCG2, PIK3CG, THSD7A, DYSF, EGFR, DPP4, HTR7, CD38, THBS4, GPR4, MAPKAPK3, COL15A1, GATA6, FZD5, SGPL1, EMP2, F2RL1, OSR1, DDAH1, FGG, PDGFA, SEMA3E, WDR35, C3AR1, MEOX2, ATF2, HK2, APOD, ADRA2A, CX3CL1, NCKAP1L, EDNRA, CALCRL, FGF18, CD40, PIK3R2, LTBP1, STK4, LIF, NRP2, LRP2, ITGA1, APLN, HRH1, DBH, DNM2, CHGA, DLL1, STRA6, RUNX1, ALDH1A2, KCNA5, P2RX1, ECM1, HOXA3, HIF1AN, TNFSF12, FN1, AHR, NR2E1, ADORA2B, PIK3R3, TAB1, PRRX1, HTR1D, MAPK11, AQP1, LEPR, CXCL10, BMP6, SERPINE1, ZFPM2, PTAFR, ROBO2, PDGFRB, STC1, LOX, TGFBR3, IL1A, CCR2, FYN, EDN3, VEGFC, NRCAM, HIPK2, LRG1, AKT1, GPC3, ANTXR1, PDGFRA, ADM, PCSK5, CCBE1, SEMA4A, PTGS2, BMP7, EDN2, CLDN1, HAS2, SIX1, ITGB3, CDC42, VTN, SEMA5A, BMP2, BMP4, MCAM, RSPO3, COL5A1, DCN, COL8A2, ITGB8, PRICKLE1, CX3CR1, HPGD, VEGFA, LAMA1, MMP19, SOCS3, EPHB2, WNT7B, CYFIP2, AGTR2, BGN, COL1A2, PRKCA, BAIAP2, GHSR, EIF2AK3, COL8A1, IL6, SEMA3C, EPHX2, DLL4, GATA4, HMGA2, NR2F2, PRDM1, TMEM100, FGFBP1, ANGPT2, ESM1, ITGA4

Table 2

LRRTM2, LRFN5, GAD1, GAD2, KRAS, GABRB2, GABRB3, GABRA5, BSN, CNTN2, GABRA2, CDH10, GAP43, ERC2, FEZF2, ASCL1, GABRB1, NPAS4, DLX2, GABRA1, DLX1, CNTNAP4, LRRTM1, SLITRK1, JAKMIP1, KCND3, NRXN1, CNR1, KCND2, LHX6, GABRA4, CALB1, ARX, PTPRO, SEZ6L2C, GABRG2, SLC32A1, PLCL1, NKX2.1, ERBB4, NRG1, PAX6, LHX1, SLC32A1, ISL1, CXCR4, CDK5R1, FOXG1, EMX2, ETV1, GABRA1, NETO1

Supplementary Figure 8: Gene expression overlap between mouse and human periventricular endothelial cells. (a, b) Venn diagrams depict a significant overlap of genes related to categories - blood vessel development (a) and GABA pathway (b) between mouse periventricular endothelial cells (PV ECs) and human PV ECs. Common genes that are expressed in mouse PV ECs and human PVECs in (a) and (b) are listed in Table 1 and Table 2 respectively.

Supplementary Figure 9:

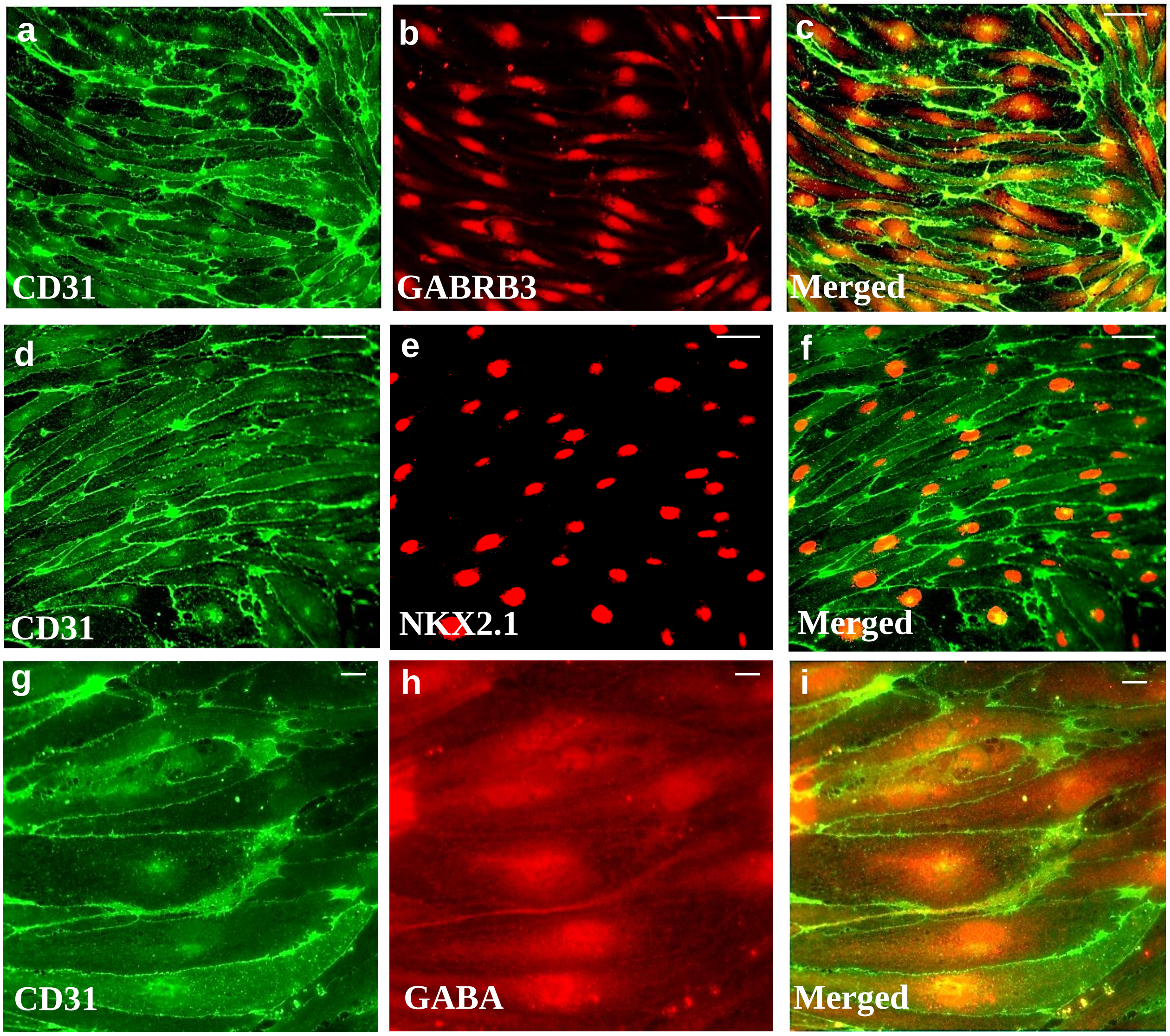

Supplementary Figure 9. Periventricular endothelial cells derived from human embryonic stem cell line H1. All H1-derived periventricular endothelial cells co-express the endothelial marker CD31 with periventricular markers GABRB3 (a-c), NKX2.1 (d-f), and GABA (g-i). Scale bars: a, 100 µm (applies to b-f); g, 50 µm (applies to h, i).

Supplementary Figure 10 :

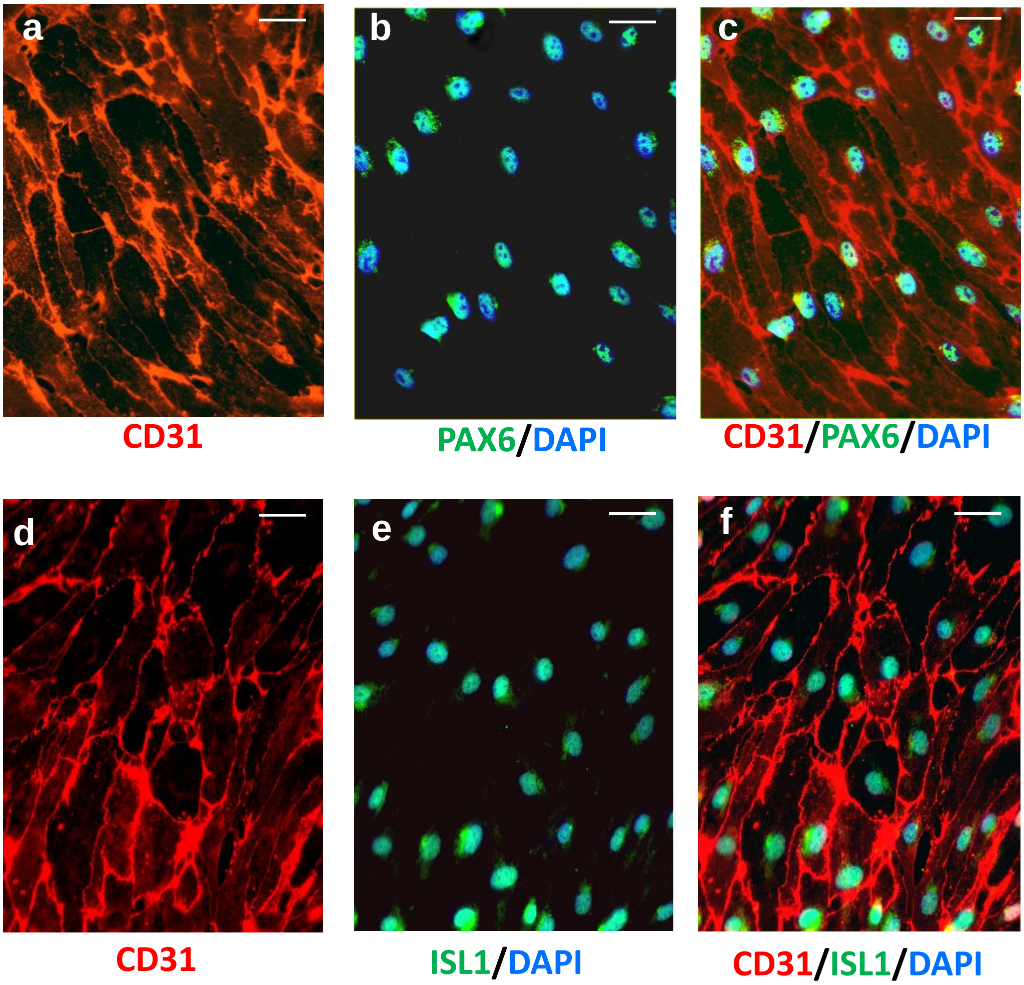

Supplementary Figure 10: Expression of PAX6 and LIM. Double-immunostaining shows that human periventricular endothelial cells express PAX6 (a-c) and LIM homeobox protein ISL1 (d-f). Scale bar: a, 100 μm (applies to b-f).

Supplementary Figure 11:

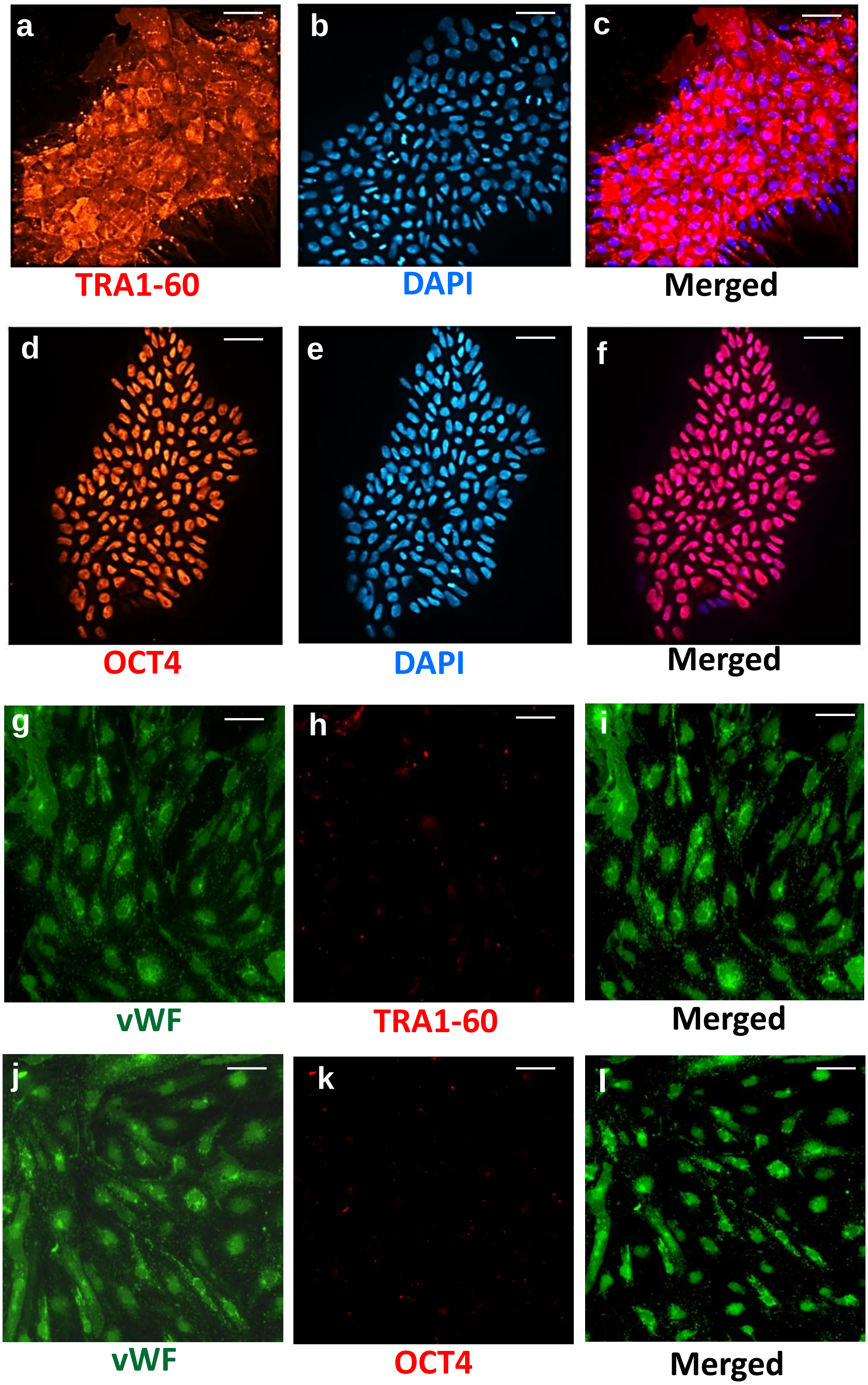

Supplementary Figure 11: **Expression of pluripotency markers.** Undifferentiated H9 ES cells express the pluripotency markers TRA1-60 and OCT4 (a-f) while human periventricular endothelial cells do not express these markers (g-l). Scale bar; a, 100 µm (applies to b-l)

Supplementary Figure 12:

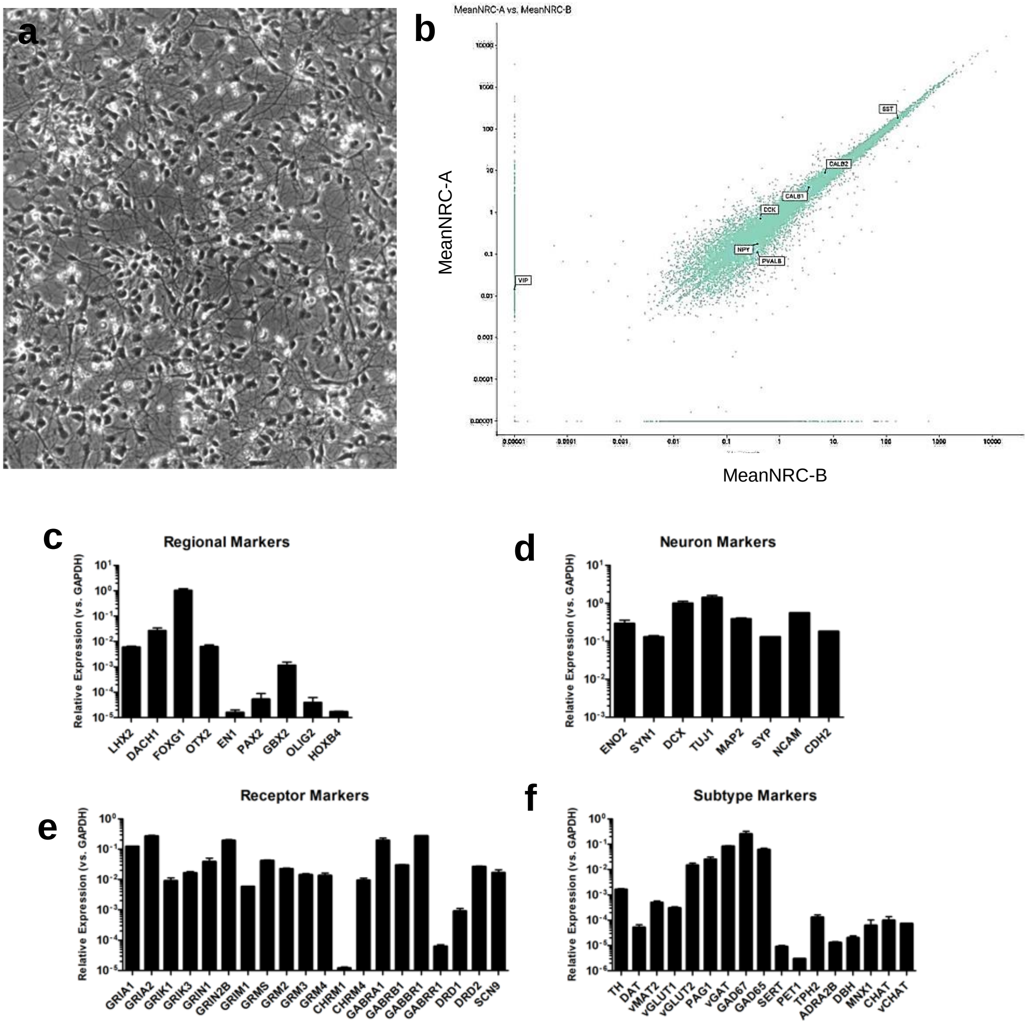

Supplementary Figure 12: Morphology and gene expression profiles of human GABAergic interneurons. (a) Phase contrast image of GABAergic interneurons that were cultured for 2 weeks after thawing. (b) RNA-seq data showing expression levels of interneuron-subtype specific markers: parvalbumin (PVALB), calbindin1 (CB1), calbindin2 (CB2), calretinin (CR), somatostatin (SOM), vasointestinal protein (VIP), neuropeptide Y (NPY) and cholecystokinin (CCK). RNA-Seq libraries were made and run on Illumina HiSeq 2000 instrument. (c-f) Relative gene expression of different regional and neural subtype markers in GABAergic interneurons.
[Personal communications; Cellular Dynamics International, Madison, WI]

Supplementary Figure 13:

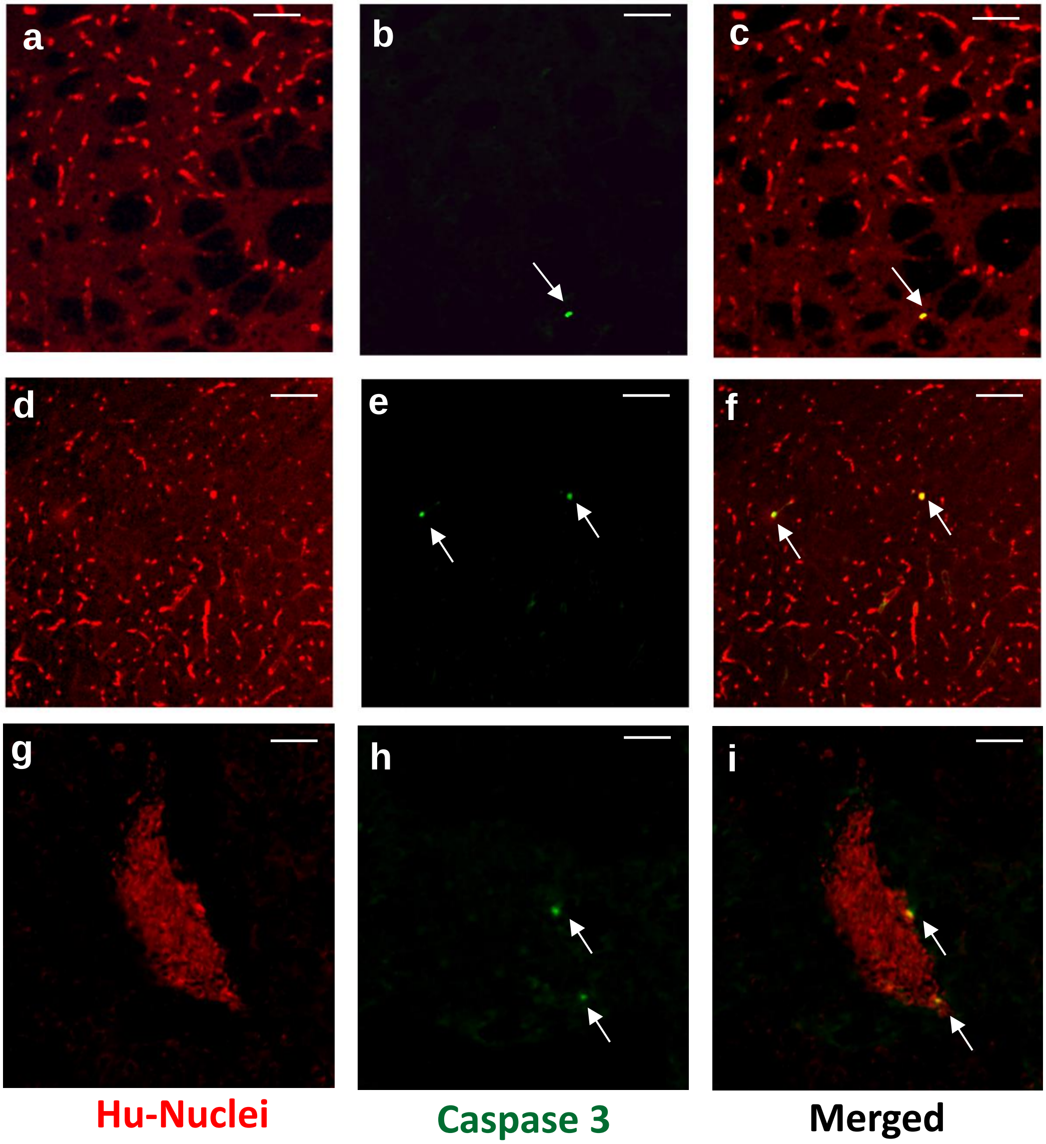

Supplementary Figure 13: Caspase staining in grafted cells. (a-i) Co-labeling with anti-human nuclei and anti-active caspase 3 antibodies in striatum (a-c) and cortex (d-f) of interneurons + periventricular endothelial cells transplanted adult NOD-SCOD brain, and in striatum (g-i) of interneurons-only transplanted adult NOD-SCID brain. Very few grafted cells were positive for caspase 3 staining (white arrows), showing that most grafted cells survived in the host brain. Scale bar: a, 100µm (applies to b-i).

Supplementary Figure 14:

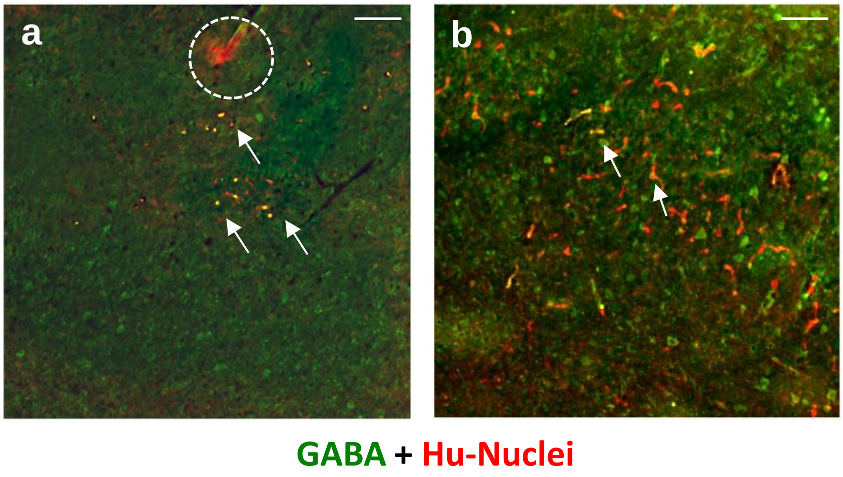

Supplementary Figure 14: Cell migration in interneuron-only and co-transplanted *Gabrb3*^{ECKO} cortex. (a, b) Double staining with anti-human nuclei (red) and anti-GABA (green) antibodies in the cortex of transplanted brains. (a) In interneuron-only *Gabrb3*^{ECKO} transplanted brain, human nuclei^{+}/GABA^{+} transplanted cells (white arrows) remain close to the grafted site (marked by dotted area). (b) In co-transplanted *Gabrb3*^{ECKO} brain, cells show widespread migration (white arrows) in the cortex. Scale bar: a, 100µm (applies to b).

Supplementary Figure 15:

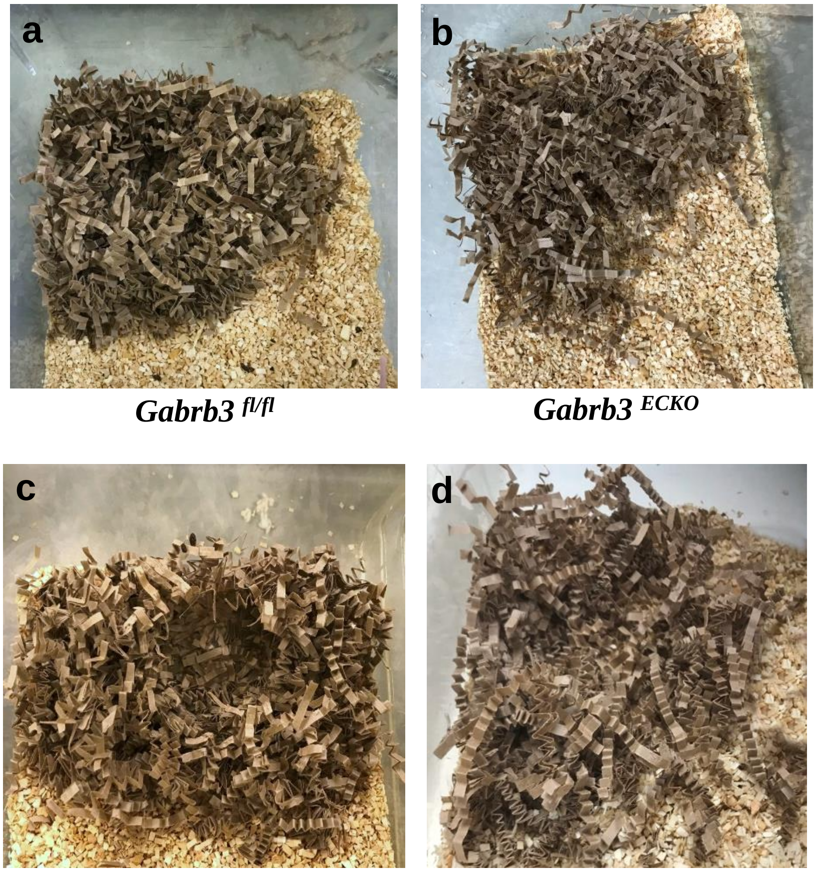

Supplementary Figure 15: Rescue of nest building behavior in co-transplanted *Gabrb3*^{ECKO} mice. Nest building ability of transplanted mice were evaluated after one month of transplantation. Mice were provided with 8 grams of shredded paper as nesting material, and nesting was assessed after 24 hours according to a five-point scale. (a) A compact, high quality nest built by sham control *Gabrb3*$^{fl/fl}$ mice. (b) Poor nest formed by *Gabrb3*^{ECKO} mice. (c) Interneurons + periventricular endothelial cells co-transplanted *Gabrb3*^{ECKO} mice built high quality nests after one month. (d) Interneuron-only transplanted *Gabrb3*^{ECKO} mice built poor nests,, just like *Gabrb3*^{ECKO} mice. This shows that poor nesting behavior of *Gabrb3*^{ECKO} mice could be rescued within one month by co-transplantation of interneurons + periventricular endothelial cells, but not by transplantation of interneurons alone. PV ECs = periventricular endothelial cells

Supplementary Figure 16:

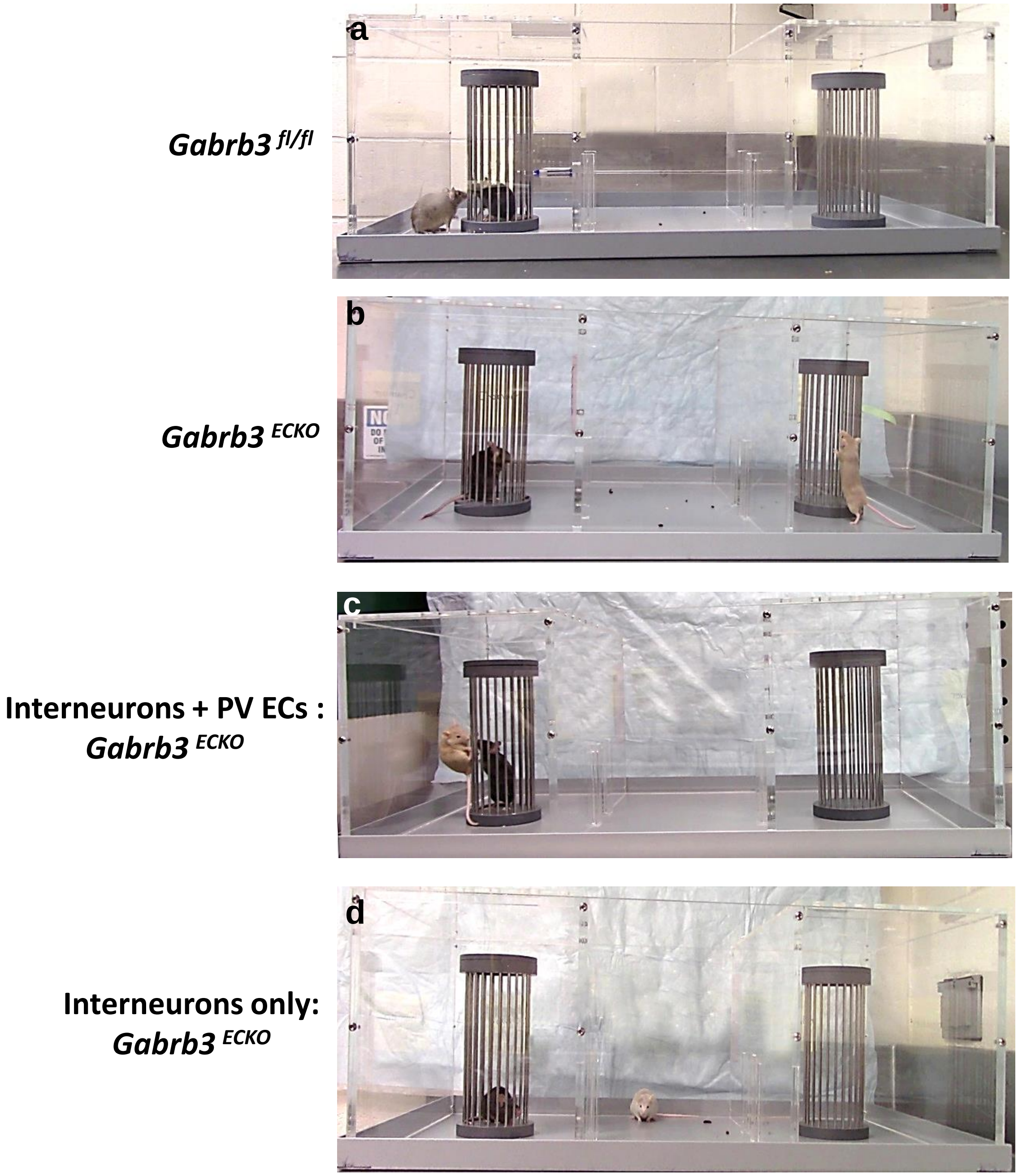

Supplementary Figure 16: Rescue of social-interaction deficit in co-transplanted *Gabrb3^{ECKO}* mice. (a) In a three-chambered social approach task, control *Gabrb3$^{fl/fl}$* mouse spent more time with stranger mouse than with an in-animate novel object. (b) *Gabrb3 ^{ECKO}* mice showed no extra preference for stranger mouse, and spent similar time exploring both the chambers. (c) Co-transplanted *Gabrb3 ^{ECKO}* mice showed significantly higher preference towards interacting with stranger mouse. (d) Interneuron-only transplanted mice did not show a preference for stranger mouse, and spent more time investigating the inanimate cage. They also spent a considerable amount of time in the middle chamber. PV ECs = periventricular endothelial cells

Supplementary Figure 17:

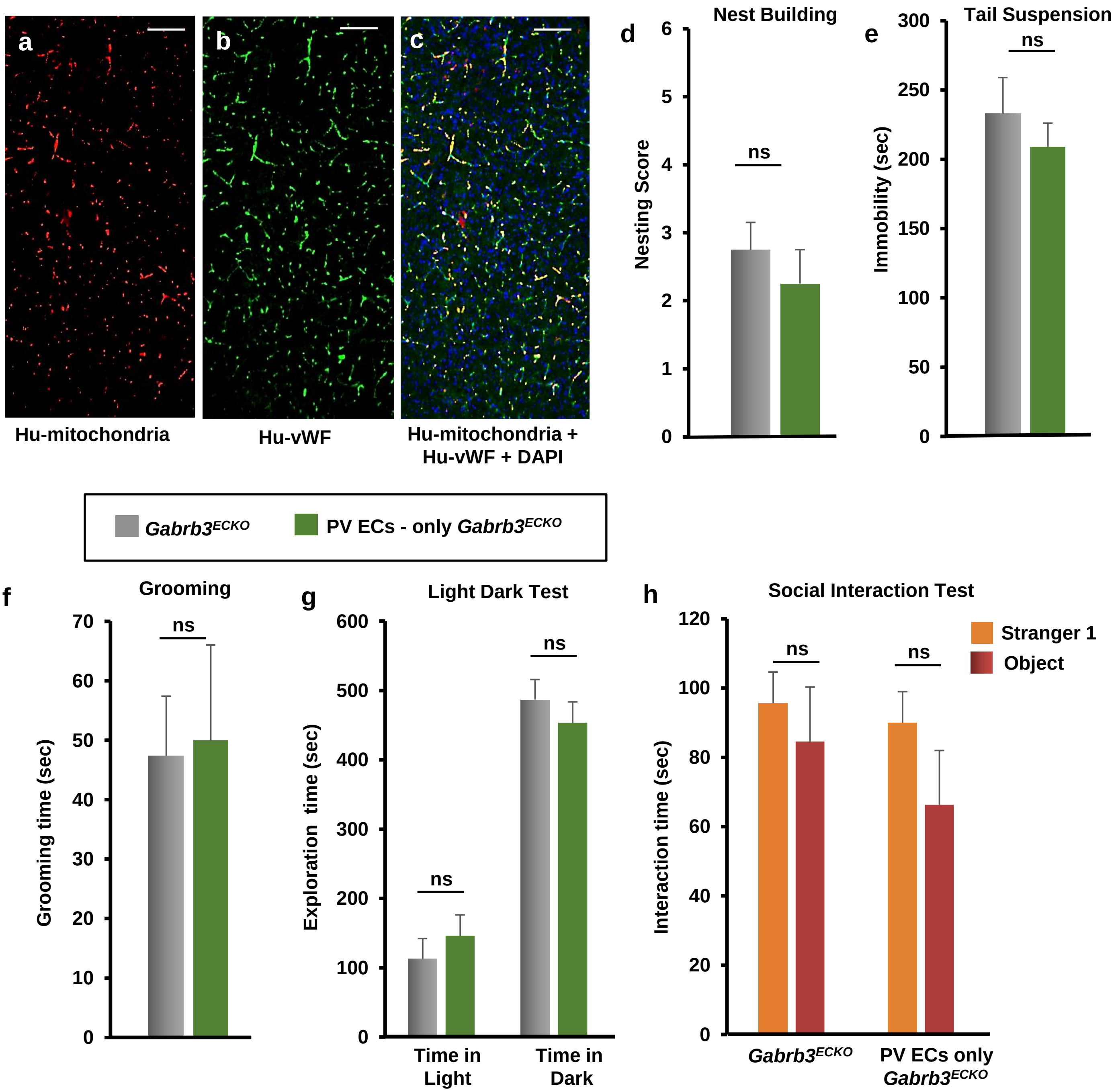

Supplementary Figure 17: Behavioral deficits in periventricular endothelial cell - only transplanted *Gabrb3^{ECKO}* mice. (a-c) Double staining with anti-human mitochondria (red) and anti-human vWF (green) antibodies in the cortex of periventricular endothelial cell-only *Gabrb3*^{ECKO} transplanted brains, showing widespread migration of transplanted endothelial cells in the cortex. (d-h) Comparison of behavioral tests performed in periventricular endothelial cell (PV EC) - only transplanted *Gabrb3*^{ECKO} mouse vs *Gabrb3*^{ECKO} mice, one month after transplantation. Periventricular endothelial cell - only transplanted mice continued to show behavioral deficits that is comparable to *Gabrb3*^{ECKO} mice (d-h) and interneurons only - *Gabrb3*^{ECKO} mice (Fig. 5). They had poor nest building ability (d), long immobility time in tail suspension test (e), high self-grooming time (f), high exploration time in dark in light-dark box test assay (g), and poor social interaction ability (h). In each case, data represents represents mean ± S.D (n=4; P>0.05 is not significant (ns), Student's t-test). Scale bar: a, 100μm (applies to b, c).
